# Current Non-Metal Nanoparticle-Based Therapeutic Approaches for Glioblastoma Treatment

**DOI:** 10.3390/biomedicines12081822

**Published:** 2024-08-11

**Authors:** Agata M. Gawel, Anna Betkowska, Ewa Gajda, Marlena Godlewska, Damian Gawel

**Affiliations:** 1 Department of Histology and Embryology, Medical University of Warsaw, Chalubinskiego 5, 02-004 Warsaw, Poland; agata.gawel@wum.edu.pl; 2 Department of Cell Biology and Immunology, Centre of Postgraduate Medical Education, Marymoncka 99/103, 01-813 Warsaw, Poland; anna.betkowska@cmkp.edu.pl (A.B.); ewa.gajda@cmkp.edu.pl (E.G.); marlena.godlewska@cmkp.edu.pl (M.G.)

**Keywords:** non-metal nanoparticles, glioblastoma, novel therapeutic strategies

## Abstract

The increase in the variety of nano-based tools offers new possibilities to approach the therapy of poorly treatable tumors, which includes glioblastoma multiforme (GBM; a primary brain tumor). The available nanocomplexes exhibit great potential as vehicles for the targeted delivery of anti-GBM compounds, including chemotherapeutics, nucleic acids, and inhibitors. The main advantages of nanoparticles (NPs) include improved drug stability, increased penetration of the blood–brain barrier, and better precision of tumor targeting. Importantly, alongside their drug-delivery ability, NPs may also present theranostic properties, including applications for targeted imaging or photothermal therapy of malignant brain cells. The available NPs can be classified into two categories according to their core, which can be metal or non-metal based. Among non-metal NPs, the most studied in regard to GBM treatment are exosomes, liposomes, cubosomes, polymeric NPs, micelles, dendrimers, nanogels, carbon nanotubes, and silica- and selenium-based NPs. They are characterized by satisfactory stability and biocompatibility, limited toxicity, and high accumulation in the targeted tumor tissue. Moreover, they can be easily functionalized for the improved delivery of their cargo to GBM cells. Therefore, the non-metal NPs discussed here, offer a promising approach to improving the treatment outcomes of aggressive GBM tumors.

## 1. Introduction

Currently, there is an observed intensification of attempts aimed at developing new advanced molecular systems to increase efficiency and precision in the delivery of therapeutics to cancer cells. These strategies are especially needed in the treatment of poor-prognosis cancers, including glioblastoma multiforme (GBM) [1]. Glioblastoma multiforme is a grade IV tumor that is recognized as one of the deadliest cancers, with a 5-year survival rate of ~5.5% [1]. GBM is more common in males, who also have a worse prognosis and a survival disadvantage, in comparison to female patients [2,3]. The average age at diagnosis is 64 ± 12.4 years [4]. 

**Figure 1:**
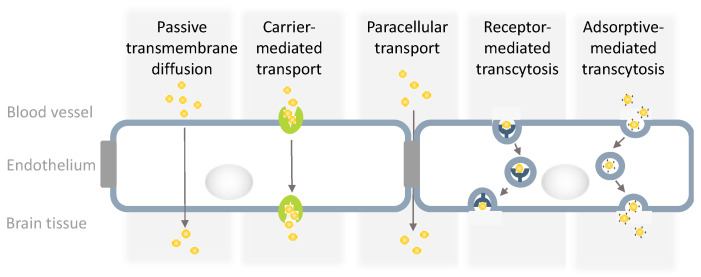
Schematic representation of transport pathways of nanoparticles across the blood–brain barrier (modified from [6]).

A significant obstacle in GBM treatment is the blood–brain barrier (BBB), which acts as a highly selective and efficient physiological guarding system preventing plasma components, including blood cells, pathogens, and toxins, but unfortunately also therapeutic agents, from entering the brain. The regulation of transport across the BBB involves various junction molecules, endothelial and pericyte transporters, as well as perivascular transport [5] (Figure 1). Therefore, the overall unique mechanisms of action of the components of the BBB guarantee its protective role and, at the same time, limit drug passage into the central nervous system (CNS). 

Due to the low survival rate of patients with GBM and the lack of effective treatment methods, novel strategies are being sought. One of the promising anticancer strategies that allows both drug delivery and better diagnostics involves the use of nanoparticles (NPs). The nanocarriers currently being studied are generally divided into metal- (already described by Gawel et al., 2021 [7]) and non-metal-based groups (discussed here). NPs offer various unique features, including high cargo capacity and the ability to target selected cells, when decorated with appropriate ligands [7,8,9], thermodynamic stability, low toxicity, and sustained drug release. Importantly, a large fraction of NPs are capable of binding both hydrophilic and hydrophobic compounds [9,10,11]. Also, NPs offer increased solubility and stability in terms of a wide range of agents, including drugs or peptides enclosed within the nanophases, which is achievable due to the maintenance of a stable pH. This is particularly important for drugs, such as temozolomide (TMZ), which is an alkaline prodrug belonging to the class of imidazotetrazinones that is recommended as a first-line drug in the treatment of high-grade GBM.

**Figure 2:**
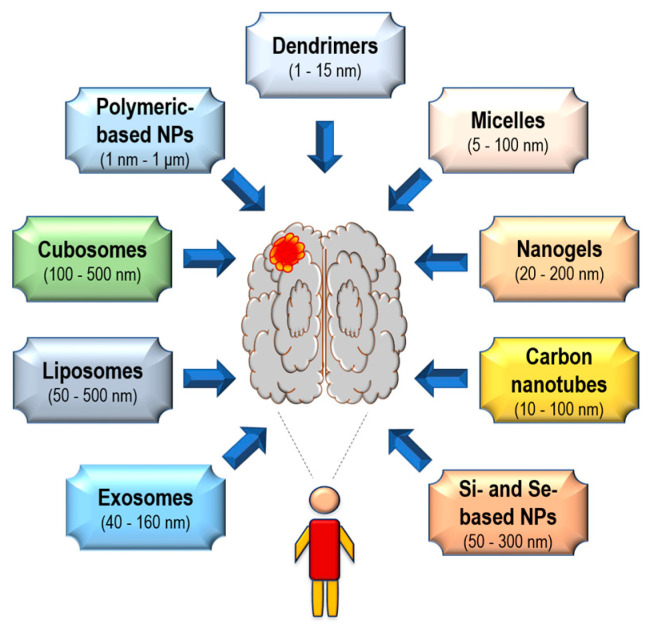
Graphical summary of the discussed non-metal-based nanoparticles (NPs) considered for the treatment of glioblastoma. In parentheses, the average size of the NPs is indicated. Si—silica; Se—selenium.

Here, we provide a summary of selected recent advancements in the treatment of GBM tumors, using non-metal-based nanoparticles (N-M NPs). The majority of data addressed in the present narrative review were published in the years 2018–2024 and were acquired using web-based biomedical literature search engines. The main N-M NPs discussed here are: (i) exosomes; (ii) liposomes; (iii) cubosomes; (iv) polymeric NPs; (v) micelles; (vi) dendrimers; (vii) nanogels; (viii) carbon nanotubes; and (ix) silica and selenium-based NPs (Figure 2). 

## 2. Exosomes

Exosomes have lately been designated as a promising tool in the therapy of various cancers, including GBMs. These 40–160 nm spherical vesicles that contain a lipid bilayer membrane were first reported nearly 40 years ago and are one of three types of cell-derived extracellular vesicles, next to microvesicles and apoptotic bodies [12,13]. 

Exosomes can be released naturally by almost all kinds of eukaryotic cells, including macrophages, erythrocytes, platelets, lymphocytes, fibroblasts, and dendritic cells, as well as tumor and mesenchymal stem cells (MSCs), or they can be developed artificially [13,14]. MSC-derived exosomes are the most widely researched exosomes, as they present low immunogenicity due to the lack of molecules, such as co-stimulatory CD86 or CD80 proteins, or class I and II human major histocompatibility complex (MHC) proteins [13].

Exosomes are predominantly produced via endosomal-like formation. This process involves cell membrane invagination and multivesicular body formation, followed by fusion with the cell membrane and release from the cell [15]. The primary function of exosomes is to aid intercellular communication by exchanging molecularly “encoded” information between cells [16]. In tumor-derived exosomes, this kind of communication may promote tumor microenvironment (TME) regulation, angiogenesis, invasion, and metastasis [17]. 

Due to their natural occurrence in the human body, exosomes exhibit low biotoxicity and immunogenicity, which makes them particularly interesting candidates for drug delivery [16,18,19]. Most importantly for their potential use as nanovehicles, they can carry multiple molecules, including proteins, lipids, nucleic acids (RNAs; DNAs) inside their aqueous core or within the lipid membrane bilayer [20,21]. Crucially for GBM treatment, exosomes can bypass the BBB and, thus, effectively deliver drugs to tumor cells [18,22]. Interestingly, exosomes use several pathways for traversing the BBB, with transcytosis suggested as the predominant mechanism [23].

Overall, recent studies focus on two major aspects of exosome use in GBM treatment: (i) exosomes as potential nanovehicles for drug or nucleic acid delivery; and (ii) combinatory usage of exosomes, drugs, and/or RNAs as a treatment strategy.

### 2.1. Exosomes as Drug or Nucleic Acid Delivery Systems

The therapeutic potential of drug-loaded exosomes has been investigated for some of the most frequently used anticancer drugs, including doxorubicin (DOX) [24,25]. The main limitation of using DOX in GBM therapy is its limited ability to traverse the BBB [23]. To enhance its delivery through the BBB into the GBM tumor site, the use of exosomes loaded with DOX (named ENPDOX) has been proposed. Namely, in a study by Zhang et al. (2021), it was shown that the systemic administration of mouse brain endothelial cell-derived ENPDOX to GBM-bearing mice mitigated the proliferation of GBM cells in vivo, increased their apoptosis, and prolonged the overall survival of the tested mice [24]. 

Another interesting approach in regard to the exosome-based delivery of drugs to cancer cells, involved the use of paclitaxel (PTX)-loaded carriers. PTX has been widely used in cancer treatment, including in the treatment of brain tumors; however, its ability to cross the BBB and, therefore, target GBM cells, remains limited. A study performed by Salarpour et al. (2019) showed that PTX-loaded exosomes, derived from the U-87 GBM cell line, produced a significantly stronger cytotoxic effect in comparison to a free PTX solution. The observed survival of the treated cells was reduced to 60% [26]. An antitumor effect of PTX-loaded exosomes was also confirmed by Duhamel et al. (2018), but in this case, PTX was used as an enhancer of the secretion of antitumoral factors by macrophages. PTX-activated macrophages produced exosomes capable of suppressing the growth of glioma cells, especially when the treatment was additionally combined with the invalidation of the proprotein convertase 1/3 [27]. 

Other examples of successful loading of exosomes with anticancer drugs include the use of atorvastatin (a commonly used lipophilic statin with pro-apoptotic activity) or selumetinib (a novel anticancer drug) [28,29]. Both of these approaches resulted in an antitumor effect and confirmed the exosomes’ potential as drug-delivery tools for the treatment of GBM.

Since exosomes have been proven to be successful carriers of nucleic acids, researchers have been exploring the therapeutic potential of RNA-loaded exosomes, particularly microRNA-loaded exosomes, to regulate tumor biology by altering the expression of cancer-related genes [30]. MicroRNAs (miRNAs; miRs) are small RNA molecules responsible for governing numerous key molecular pathways [31]. They have been found to act as either oncogenes or tumor suppressors. Zhang et al. (2021) reported that miR-29a-3p, which is highly expressed in normal brain tissue, in contrast to GBM cells, inhibits the formation of vasculogenic mimicry and the migration of glioma cells. Therefore, miR-29a-3p-overexpressing exosomes can potentially support anti-vascular endothelial growth factor (VEGF) therapy and limit the blood supply, consequently restricting the growth of the tumor [32]. Another promising miR molecule for application in the therapy of GBM is miR-584-5p. Glioma cells treated with MSC-derived exosomes transfected using miR-584-5p were found to have a restricted invasive and proliferative potential, alongside an increased apoptotic ability. Additionally, exposure to these exosomes reduced the expression of matrix metalloproteinase-2 (MMP-2), which promotes carcinogenesis, and, therefore, miR-584-5p was suggested to suppress the metastasis of glioma cells [33]. Similarly, MSC-derived exosomes containing miR-133b, which was downregulated in glioma cells, inhibited the proliferation, invasion, and migration of glioma cells [34].

Following the possibility of using miRs as therapeutic/diagnostic targets in treatment strategies for gliomas, exosomal long non-coding RNAs (lncRNAs), or small interfering RNAs (siRNAs), have also been considered. Promising prospects for future therapy include the glioma-promoting ROR1-AS1, or chemotherapy resistance-promoting SBF2-AS1 lncRNAs, alongside siRNA targeting the *FGFR3*-*TACC3* fusion gene, important for promoting gliomagenesis [35,36,37]. Another example of a key regulator of GBM progression, which is effectively targeted using exosome-encapsulated siRNAs, is the Myc oncogene (a crucial signaling pathway regulator). Research carried out by Haltom et al. (2022) showed that using Myc-targeting siRNA, carried inside human bone marrow-derived MSC exosomes, inhibited GBM cell proliferation and effectively suppressed tumor growth [38].

### 2.2. Combinatory Use of Exosomes, Drugs, and Other Therapeutic Agents 

Since exosomes exhibit the ability to carry both small molecules and macromolecules, numerous research efforts have focused on loading these NP-based vesicles with multiple treatment agents to enhance their delivery into tumor cells [39]. For example, it has been shown that the usage of blood exosomes for the combined delivery of cytoplasmic phospholipase A2 siRNA and metformin successfully reduced tumor growth by affecting GBM energy metabolism [40]. Another combinatory strategy that has been proposed for GBM treatment involves using a cocktail of synergistically acting therapeutic agents that together promote apoptosis [41]. A study by Rahmani et al. (2023) employed MSC-derived exosomes loaded with two apoptosis-inducing agents (cytosine deaminase and miR-34a). These exosomes presented anti-epidermal growth factor receptor (EGFRvIII) antibodies on their surface, which enabled them to precisely target EGFRvIII-positive GBM cells and enhanced their apoptotic rate [41].

To improve the therapeutic outcomes of drug-loaded exosomes, several other studies have also explored the idea of functionalizing exosomes by conjugating them with functional protein ligands, antibodies, or peptides [41,42,43,44]. One such example showed that exosomes derived from a mouse macrophage cell line loaded with curcumin (a polyphenol exhibiting pro-apoptotic and pro-proliferative properties) and superparamagnetic iron oxide nanoparticles (SPIONs), and subsequently conjugated with the neuropilin-1 (NRP1)-targeting peptide, are effective theranostic agents in terms of gliomas [45]. Interestingly, curcumin and SPIONs were proven to individually reduce the rate of tumor growth; however, their co-delivery was found to be more effective. Moreover, it was reported that modified exosomes were not only able to effectively cross the BBB, but also successfully suppress the growth of tumors in vivo, thus extending the survival of U251 GBM-bearing mice [45]. 

### 2.3. Advantages and Limitations of Exosome Usage as Drug-Delivery Vehicles

Exosomes can be considered as highly promising nanocarriers of therapeutic agents in the treatment of GBM, predominantly due to their natural ability to cross biological barriers, most importantly the BBB [18]. The main advantages of exosomes as nanovehicles, in comparison to their synthetic counterparts, are low immunogenicity and toxicity, as well as high biocompatibility. Moreover, their enrichable structure enables higher tumor specificity, thus enhancing the delivery of drugs into cancer cells [13].

Nevertheless, naturally-derived exosome-based drug-delivery systems have some limitations. The main disadvantages of cell-derived exosomal nanovehicle technologies are the low yield of nanoparticles, as well as the cost and non-standardized isolation methods. Altogether, this affects the scalability of the technology and could be the reason behind the limited clinical application of exosomes [46,47]. Hence, more research efforts have been focusing on using artificially developed exosomes. However, this type of nanocarrier could encounter other challenges, such as higher toxicity and rapid clearance [13]. Some of the other drawbacks of natural exosomes in GBM treatment are the targeting efficiency and early drug release in circulation. Fortunately, such limitations can be overcome by the functional modification of exosomes, for example with redox-response oligopeptides [48]. 

Overall, exosomes hold great applicative potential as cell-derived N-M-based NPs for GBM treatment. With further scalability improvements and more exosome-focused research, these nanovehicles could serve as an effective drug-delivery nanoplatform.

## 3. Liposomes

Liposomes are non-toxic, artificial vesicles, with a spherical structure, characterized by high stability and biodegradability. Therefore, they have been widely considered as promising candidates for the delivery of chemotherapeutics to GBMs. Their main advantage related to treatment results from the overall enhanced anticancer effectiveness of the transported drugs [49]. The transport of liposomes across the BBB is possible through: (i) adsorptive-mediated transcytosis; (ii) receptor-mediated transcytosis; and (iii) carrier-mediated transcytosis [50]. Liposomes are often adapted by the encapsulation of hydrophilic molecules or the functionalization of their outer surface using, for e.g., peptides, ligands, tri-branched glucose, apolipoprotein E (ApoE), or polyethylene glycol (PEG) [51,52]. Their functionalization enables them to overcome the BBB and increase the cargo uptake by GBM cells [53]. Similar to other NPs, they may be delivered to GBM using the intranasal route, which enables the circumvention of the BBB [54,55]. 

### 3.1. Liposomes as Chemotherapeutic Carriers and Their Modification

Drug-loaded liposomes are presently one of the most extensively studied nano-based treatment strategies for GBM. Liposomes encapsulating PTX, DOX, and TMZ have been shown to effectively access malignant cells, ensuring enhanced antitumor efficacy, which resulted in improved survival in the murine model [56,57,58]. To increase the effectiveness of treatment strategies, the co-loading of drugs with other compounds into liposomes is being explored. The general expectation in regard to such carriers is that they will induce synergistic tumor suppression. An example of such a multicomponent tool are liposomes loaded with cisplatin (CSP) or TMZ, as well as fisetin (a flavonoid with antioxidant properties) and a pro-apoptotic peptide targeting mitochondria [59,60]. 

Also, modifications of liposomes have been shown to increase the accessibility and cytotoxic properties of the drugs enclosed within the carrier. The coating of liposomes using the cell-penetrating peptide R8 has been shown to greatly improve their ability to deliver DOX to U-87 cells [61], while the PEGylation of liposomes carrying TMZ elongated their plasma circulation time and the brain bioavailability of the drug [62]. Gharferi et al. (2022) reported that PEGylated liposomes dual loaded with DOX and carboplatin not only presented preserved stability, but also limited the side effects of the drugs (e.g., reduced liver cell necrosis), in the in vivo rat model. Importantly, the survival of the rats treated with the modified liposomal carrier was elongated by 21 days, in comparison to the non-treated group, and 9 days when compared to the group treated with free drugs [63]. 

### 3.2. Liposomes as Carriers of Nucleic Acids and Inhibitors

Liposomes loaded with nucleic acids, including both miRs and siRNAs, have received special attention in recent years [64,65]. Certain miRs, such as miR-92b, have been indicated as promising anti-GBM therapeutic targets, as their high expression is observed in malignant brain cancer. Grafals-Ruiz et al. (2020) reported that functionalized gold–liposome NPs, serving as platforms for the delivery of RNA oligonucleotides, may inhibit the expression of miR-92b, which targets the tumor suppressor gene *FBXW7* [66]. In the following studies, they tested the systemic administration of the liposomal miR-92b inhibitor in GBM xenograft mice and showed a reduction in the tumor volume and weight [67]. MiRs encapsulated in liposomes, including miR-603, have been reported to be promising radiosensitizers of GBM cells [64]. Moreover, the application of RNA as a therapeutic factor of multicomponent carriers was tested. An example of such a tool is a nanocomplex built from dual-modified cationic liposomes, a lipoprotein receptor-related protein, and anticancer agents (survivin siRNA and PTX), which was capable of actively targeting, imaging, and treating CD133+ glioma stem cells (GSCs), after passing through the BBB [68]. 

At present, there is an observed strong interest by the pharmaceutical industry in the development of kinase inhibitors as a novel class of anticancer compounds. Therefore, liposomes encapsulating inhibitors open up new therapeutic possibilities. There have been studies on the targeted delivery of mTORC1/mTORC2 inhibitor-loaded liposomes to glioma cells or elongated survival after a treatment consisting of photodynamic therapy (PDT) and liposomes encapsulating lapatinib (an EGFR inhibitor) in glioma-bearing rats [69,70]. 

### 3.3. Methods for the Improved Delivery of Liposomes across the Blood–Brain Barrier

Due to the difficulties in the efficient delivery of liposomes to the target tumor cells, local and reversible opening of the BBB for the delivery of drug-carrying liposomes is being achieved through various strategies [71,72,73]. Magnetic resonance imaging (MRI)-guided low-intensity focused ultrasound, with the intravenous administration of microbubbles, together with chemotherapy (DOX or TMZ), has been confirmed to be a safe and effective method for disrupting the BBB [74]. The effectiveness of this strategy in opening the BBB for the delivery of the liposome-loaded O^6^-Methylguanine-DNA methyltransferase (MGMT) inactivator, in combination with TMZ treatment, has been established to decrease the growth of GBM and increase the survival of mice bearing TMZ-resistant tumors [75]. 

Also, the efficacy in crossing the BBB by thermosensitive liposomal systems was established in vitro. The P1NS (GBM-specific cell-penetrating peptide) and TN-C (anti-GBM antibody)-conjugated liposomal construct, carrying DOX and SPIONs, was found to effectively cross the BBB and precisely target malignant U-87 cells [76]. Apart from DOX, magnetic temperature-sensitive liposomes may carry TMZ and result in an increase in GBM cell death [77]. Temperature-sensitive liposomes have been combined with PTX and fluorophore (NIR-II) and photothermal dyes to create a nanomedicine that allows for simultaneous GBM ablation and drug release [78]. Thermosensitive liposomes have also been evaluated as delivery vehicles for boron derivatives in boron neutron capture therapy and it was determined that they enhance targeting of the tumor [79,80]. Liposomal sodium borocaptate presented superior long-term results in comparison to other boronated compounds, as it effectively restricted tumor growth in U-87 GBM-bearing SCID mice (deficient in T and B lymphocytes) [81]. 

Due to the success in the penetration of Angiopep (Ang)-2-conjugated liposome-silica hybrid NPs with attached polyacrylic acid across the BBB, liposomes encapsulating arsenic trioxide and modified with Ang-2 have also been found to be successfully delivered to the glioma tissue, resulting in an antitumor effect [82]. Similarly, Ang-2- and anti-CD133 monoclonal antibody-modified immunoliposomes loaded with TMZ were found to promote apoptosis and migration of GSCs isolated from the U-87 GBM cell line, while a study performed on mice carrying intracranial tumors revealed a reduction in the size of the tumor and prolonged survival after treatment with immunoliposomes [83]. An even more successful modification of liposomes for enhanced transcytosis and GBM targeting can be achieved using the ApoE-derived peptide. Such liposomal systems have been used to deliver TMZ and DOX to GBM cells, which resulted in an over 2-fold increase in the survival of orthotopic U-87 tumor-bearing mice treated with the ApoE–DOX liposomes, relative to the control group (treated only with PBS) [84,85,86].

To improve passage through the brain–blood-endothelial barrier, without disrupting its integrity, the functionalization of liposomes using single-domain antibodies, such as RG3, is promising. When administered to LN229 GBM cells, such a nanocomplex containing panobinostat (a histone deacetylase inhibitor) presented an antiproliferative effect [87].

### 3.4. Novel Approaches to Liposomes

Nanoliposomes are stable nanoscale bilayer lipid vesicles that have a large surface area and are favorable for the transport of both hydrophilic and hydrophobic complexes [88,89,90]. Nanoliposomes carrying curcumin, which were further modified with a rabies virus glycoprotein derivative, have been proposed for targeted treatment, as they extended the survival of the tested mice by 10 days [91]. 

Microfluidic-derived docosahexaenoic acid liposomes [92], transferosomes [93], cholesterol-rich nanoemulsion [94], or cerasomes (a liposomal nanohybrid) that present superior morphological stability, are also considered as a promising nanotool for the transportation of drugs (e.g., docetaxel) [95]. 

An interesting nanoformulation possessing numerous advantageous properties, including stability, improved tumor penetration, and low toxicity to normal cells, are biomimetic liposomes, which may be implemented in phototheranostics [96]. Additionally, a strategy that enhances liposomal anticancer properties and improves brain targeting is an approach that involves coating them with a biomolecular corona built from a layer of plasma proteins [97].

### 3.5. Liposomes in Clinical Studies

Importantly, the promising properties of liposomes in in vitro and in vivo studies have led to the initiation of clinical trials (listed in Table 1). Many studies were or are conducted on PEGylated liposomal carriers. A study, currently in progress, involves the trial on PEGylated liposomal DOX (NCT06356883) administered to patients with recurrent GBM. In contrast, an example of a terminated study is the phase I study (NCT03119064) that was conducted to assess the effectiveness of PEGylated nanoliposomal irinotecan and TMZ in patients with recurrent GBM. Twelve patients with confirmed progression of GBM, who had already undergone radiation and received TMZ, were enrolled in the trial. After treatment with the nanoplatform, the median progression-free survival (PFS) was found to be 2 months (for 10 of the patients). The trial was concluded earlier than expected, as the effectiveness of the response was found to be 0/12 and side effects, including diarrhea or neutropenia, occurred in the patients [98].

An alternative phase I trial (NCT03603379), completed in 2020, involved testing the effect of DOX-carrying immunoliposomes that had been modified to target EGFR in patients with a relapse and EGFR-amplified GBM. Nine patients were enrolled in the trial, seven out of whom received the modified immunoliposomes as second-line treatment and two as fourth-line treatment. The median PFS and overall survival were 1.5 and 8 months, respectively. Notably, one patient had a PFS of 16.4 months post-immunoliposome therapy. Most patients did not experience any side effects from the treatment [99]. The concentration of DOX in GBM tissue was found to range from 180 to 3730 ng/g tumor (assessed for three patients), while in the cerebrospinal fluid, the maximum concentration of DOX was 0.94 ng/mL. It has to be underlined that the patients received not only the anti-EGFR DOX-carrying immunoliposomes, but also other treatment strategies, such as bevacizumab; therefore, the exact clinical effect of this therapeutic modality requires further study. 

One of the most interesting recent attempts, presently recruiting patients, concerns the application of RNA–lipid particle vaccines for newly diagnosed adults with MGMT-unmethylated GBM (NCT04573140). This RNA–lipid NP aggregate vaccine is going to be administered to patients where standard therapy is not effective due to resistance to TMZ and is expected to reprogram the immunosuppressive TME.

**Table 1:** Nanoparticles investigated in clinical trials for glioblastoma treatment (registered on ClinicalTrials.gov).

Agent(s)	NP Type	Study Phase	Specific Inclusion Criteria	Study Start/ (Estimated) Completion Date	Status	Trial Number/ Reference
SGT-53 ^1^ in combination with irradiation and/or chemotherapy	Cationic liposome	I	Pediatric patient IDHs with recurrent or progressive CNS malignancies	06.2022/ 12.2023	Not yet recruiting	NCT03554707
SGT-53 ^1^ in combination with TMZ	Cationic liposome	II	Histologically confirmed glioblastoma or gliosarcoma in first, second, or third relapse	12.2014/ 11.2018	Terminated (small number of enrolled patients)	NCT02340156
NL CPT-11 ^2^	PEGylated liposome	I	Histologically confirmed intracranial malignant glioma	08.2008/ 12.2014	Completed	NCT00734682
Onivyde ^2^ in combination with TMZ	PEGylated liposome	I/II	Histologically confirmed glioblastoma multiforme	11.2017/ 04.2020	Terminated (lack of response to the studied therapy)	NCT03119064
PEG–DOX ^3^ and prolonged TMZ, in addition to radiotherapy	PEGylated liposome	I/II	Histologically confirmed glioblastoma	07.2002/ 05.2009	Completed	NCT00944801/ [100]
DepoCyt ^4^ in combination with TMZ	PEGylated liposome	I/II	Histologically confirmed glioma that failed initial surgical resection, followed by standard adjuvant therapy	09.2009/ 08.2013	Terminated (small number of enrolled patients)	NCT01044966
2B3-101 ^5^	PEGylated liposome	I/II	Histologically confirmed glioma	07.2011/ 12.2014	Completed	NCT01386580
Caelyx ^6^ liposomal DOX in combination with carboplatin	PEGylated liposome	II	Histologically confirmed glioblastoma multiforme	04.2024/ 04.2028	Recruiting	NCT06356883
EGFR(V)–EDV–DOX ^7^	Minicell	I	Histologically confirmed recurrent glioblastoma	10.2016/ 12.2019	Unknown	NCT02766699/ [101]
SGT-94 ^8^	Liposome	I	Histologically confirmed neoplasm for which no standard therapy is available	01.2012/ 12.2015	Completed	NCT01517464
C225–ILs–DOX ^9^	Liposome	I	Glioblastoma, *EGFR* amplification	11.2018/ 11.2020	Completed	NCT03603379/ [99]
Liposomal curcumin in combination with radiotherapy and TMZ	Liposome	I/II	Histologically confirmed III/IV grade glioma	03.2023/ 02.2026	Recruiting	NCT05768919
Visudyne ^10^	Liposome	I/II	Recurrent or progressive grade IV glioblastoma; standard first-line therapy (radiation, TMZ); mutant or amplified *EGFR*	01.2021/ 08.2025	Recruiting	NCT04590664
Liposomal DOX in combination with balstilimab, botensilimab, implantation of sonocloud-9	Liposome	II	Newly diagnosed pathologically confirmed glioblastoma; *IDH1*/*IDH2* wt; tumor with *MGMT* gene promoter unmethylated	01.2024/ 05.2026	Recruiting	NCT05864534
Myocet ^11^	Liposome	I	Pediatric patients having received at least one cycle of chemotherapy after radiotherapy; grade III or IV glioma	10.2010/ 04.2013	Completed	NCT02861222/ [102]
Rhenium-186 nanoliposome	Liposome	I	Histologically confirmed grade III/IV glioma	01.2023/ 01.2025	Not yet recruiting	NCT05460507
Rhenium-186 nanoliposome	Liposome	I/II	Histologically confirmed grade III/IV recurrent glioma	06.2015/ 01.2025	Recruiting	NCT01906385
RNA–lipid NP ^12^ vaccine	Liposome	I	Histologically confirmed newly diagnosed de novo glioblastoma multiforme (grade IV glioma); tumor with *MGMT* unmethylated	10.2021/ 07.2026	Recruiting	NCT04573140
DOX liposome	Liposome	I	Pediatric brain tumor	07.1999/ ND	Completed	NCT00019630

^1^ Complex of a cationic liposome encapsulating a normal human wild type p53 DNA sequence in a plasmid backbone; ^2^ nanoliposomal irinotecan; ^3^ PEGylated liposomal doxorubicin (DOX); ^4^ liposomal cytarabine; ^5^ glutathione PEGylated liposomal DOX; ^6^ PEGylated liposomal DOX; ^7^ DOX-loaded *Salmonella typhimurium*-derived EnGeneIC O-polysaccharide delivery vehicle (minicell; 400 nm) targeting epidermal growth factor receptor (EGFR)-expressing cells; ^8^ RB94 plasmid encapsulated in a liposome that is targeted to tumor cells by means of an anti-transferrin receptor single-chain antibody fragment (TfRscFv); ^9^ DOX-loaded anti-EGFR immunoliposomes; ^10^ liposomal verteporfin (benzoporphyrin derivative); ^11^ non-PEGylated liposomal DOX; ^12^ autologous total tumor mRNA and pp65 full-length lysosomal-associated membrane protein (LAMP) mRNA-loaded 1,2-dioleoyl-3-trimethylammonium-propane (DOTAP) liposome vaccine; CNS, central nervous system; IDH, isocitric dehydrogenase; MGMT, methyl guanine methyl transferase; ND, not defined; NP, nanoparticle; TMZ, temozolomide; wt, wild type.

## 4. Cubosomes

Cubosomes were developed as cubic phases for application in drug delivery. They are stable, self-structured NPs, with a small size and low viscosity, which may carry greater cargo than standard nanovehicles and can form a dispersion that requires a stabilizer and the application of external mechanical energy. The advantages of cubic nanostructures arise from their thermodynamic stability, low toxicity and, importantly, their ability to bind both hydrophilic and hydrophobic compounds [9,10,11]. They also offer improved stabilization of prodrugs, which results in their prolonged therapeutic effect [11,103,104]. 

The typical components used to synthesize these cubic nanostructures are monoolein and Pluronic F108, a tri-block co-polymer of poly(ethylene oxide) and poly(propyleneoxide) that plays the role of a stabilizer. Another alternative component may be monopalmitolein, which results in the formation of larger channels and, therefore, greater cargo capacity, stability, and half-life. The physicochemical properties of cubosomes allow them to increase the solubility and stability of a wide range of agents, including poorly water-soluble drugs, such as TMZ or DOX, as well as miRNA molecules or peptides [105,106], and then release them in a controlled and lasting manner. They also have the potential to bypass the BBB, which is probably related to the increased drug permeability [103,107]. Also, enhanced BBB penetration using cubosomes is possible through decorating them with targeting moieties, such as Tween 80 (a stabilizer) or the aforementioned Pluronics (i.e., poloxamers) [107]. Therefore, such nanoparticle molecular carriers are of great interest in the treatment of the remaining poorly curable cancers, including GBM [7,108].

Cubosomes may also be highly favorable for the co-treatment of GBM cells by loading miRs and drugs into these lipid-based liquid crystalline NPs. Gajda et al. (2020) showed that drug-sensitive and drug-resistant glioma-derived cells (A172 and T98G, respectively) treated with cubosomes loaded with miR-7-5p and chemotherapeutics (TMZ and DOX) present significantly increased apoptosis [108]. Another study confirmed the efficacy of AT101-loaded cubosomes in the therapy of GBMs, resulting in the effective entrance of the drug into GBM cells and an enhanced cytotoxic effect. AT101 (a cottonseed-derived gossypol) is characterized by low bioavailability in monotherapy; therefore, placing the drug in cubosomes supported its anticancer properties, while also overcoming the limitations resulting from the low solubility of AT101 in water-based media [109]. Finally, the functionalization of drugs carrying cubosomes using Ang-2 (for enhanced penetration of the BBB) has been reported as a beneficial method for improved uptake and higher toxicity towards U-87 GBM cells in vitro, as well as a 3-fold greater brain accumulation in C57BL/6 mice than unconjugated cubosomes [110]. 

## 5. Polymeric-Based Nanocarriers

Polymeric NPs are a broad and commonly studied group of N-M NPs. They present advantageous features including favorable drug-loading properties, the sustained release of agents at the tumor site, biocompatibility, and general non-toxicity [111]. The size of polymeric NPs ranges from 1 nm to 1 μm, which is beneficial for crossing the BBB [111]. For the treatment of GBM, the most frequently investigated subgroups of synthetic polymeric NPs include poly(lactic-co-glycolic acid) (PLGA), poly(β-amino ester) (PBAE), and poly-ε-caprolactone (PCL) NPs [112,113,114,115]. Among polymeric NPs derived from natural materials, chitosan, alginate, and gelatin are most often studied for their application in the therapy of GBM [116,117,118]. 

### 5.1. Synthetic-Derived Polymeric Nanoparticles

The main group of synthetic polymeric NPs, PLGA-based NPs, present satisfactory biocompatibility, tumor tissue specificity, and continuous release properties [112]. PLGA is a Food and Drug Administration (FDA)-approved non-toxic biodegradable polymer that can self-assemble into NPs [119,120]. PLGA NPs can both efficiently permeate the BBB or may be delivered to GBM cells via intranasal administration [120,121]. PLGA NPs effectively transport chemotherapeutics, such as DOX or PTX, to GBM cells and elongate the survival of orthotopic glioma rats >2-fold in comparison to treatment with free drugs [122]. Also, PTX-, CSP-, amrubicin- or TMZ-loaded PLGA NPs have been found to present a cytotoxic effect, reduce GBM cell progression, as well as lower the TMZ- and multidrug-resistance of GBM cells [123,124,125,126]. Apart from chemotherapeutics, PLGA NPs are applied as carriers of suberoylanilide hydroxamic acid (a histone deacetylase inhibitor with antitumorigenesis activity), disulfiram (an agent presenting anti-NF-κB and anticancer stem cell properties), etoricoxib (a nonsteroidal anti-inflammatory drug), and cannabidiol (a phytocannabinoid), to inhibit GBM progression [127,128,129]. To increase the brain-targeting properties of PLGA NPs, conjugation with transferrin has been considered [130]. The application of PLGA NPs conjugated with transferrin may also be considered for the co-delivery of bortezomib (a targeted cancer drug that downregulates the expression of MGMT) and TMZ to exert a synergistic antitumor effect [131]. The improvement of PLGA NP properties can also be achieved by coating them with chitosan [132]. PLGA/PEG NPs in the future may also be used as a matrix paste that can be inserted into the postsurgical cavity, following GBM removal [133].

PBAEs, a subclass of cationic and biodegradable polymeric NPs, possess numerous favorable features, including bioreducibility, the ability to transport specific nucleic acids (DNA, mRNA, siRNA) targeting GBM cells, as well as improved diffusion in vivo and enhanced survival in the orthotopic GBM-bearing murine model [113,134,135,136]. Interestingly, PBAE NPs have also been shown to be capable of carrying numerous siRNA molecules concurrently (i.e., the anti-GBM genes *Robo1*, *YAP1*, *NKCC1*, *EGFR*, and *survivin*), effectively targeting several targets at the same time [137]. Similarly, the simultaneous delivery of two mRNA molecules, miR-148a and miR-296-5p, using a PBAE-based system has been found to restrict tumor growth in the orthotopic model of human GBM in mice [113].

The administration of PCL (an FDA-approved biodegradable aliphatic polyester) NPs for GBM treatment is less studied [138]. Several recent reports indicate that PCL-block-PEG polymer NPs may increase the effectiveness of the delivery of anti-GBM compounds, while spray-dried (i.e., a specific three-step technique used to synthesize NPs) PCL NPs that are characterized by biocompatibility and biodegradability may effectively transport TMZ across the BBB [114,139]. 

### 5.2. Natural-Derived Polymeric Nanoparticles

A well-studied subgroup of polymeric NPs, which are derived from natural materials, are chitosan NPs. Chitosan is a linear polysaccharide that is poorly soluble in a neutral and basic pH and is considered as a neuroprotective agent [140]. Chitosan NPs can effectively carry drugs [116,124], nucleic acids, and proteins [141,142] to GBM tumor cells. Chitosan NPs loaded with mRNA, including miR-219 (a glioma tumor suppressor), have been shown to target U-87 GBM cells and reduce their survival, without affecting fibroblasts [143]. Chitosan-based NPs, such as chitosan oligosaccharide lactate NPs conjugated with folic acid–PEG have also been used for the effective delivery of CD146-specific siRNA to GSCs. Targeting CD146, a membrane glycoprotein correlating with the grade and thus the aggressiveness of glioma, has led to suppressed tumor growth in vivo [144]. 

The modification of chitosan nanoplatforms using Ang-2 and loading them with DOX can also improve their uptake by U251 GBM cells by ~2–3-fold and increase their apoptosis and necrosis by ~2-fold, relative to the cells treated with free DOX [145]. They are also being investigated as successful vehicles for bypassing the BBB, as modified chitosan-based platforms, such as transferrin-decorated chitosan NPs, may be delivered to the tumor tissue via the intranasal route [121,146,147]. This delivery method is beneficial, as it ensures high permeation of the nasal mucosa by the NPs and, therefore, has an enhanced anticancer effect.

Other natural material-based polymeric NPs include alginate (a hydrophilic anionic polysaccharide) and gelatin (a biodegradable polymer) NPs, which are characterized by sustained TMZ release and an improved cytotoxic effect [117,148,149].

### 5.3. Conjugated Polymer Nanoparticles

Interest in the application of conjugated polymer nanoparticles (CPNs; prepared from semi-crystalline semi-conducting polymers) as nanocarriers has grown in recent years, as they are capable of delivering cytostatic drugs to GBM cells and inducing their apoptosis, as well as inhibiting their proliferation and angiogenesis [150,151]. They offer excellent biocompatibility, simple synthesis, stability, and bioimaging properties, such as exceptional fluorescence brightness and photochemical stability [151,152,153]. Among CPNs, polyaniline, polypyrrole, and polyacetylene, and their derivatives, are the most studied [154]. 

CPNs play an important role in PDT as photosensitizers and may eliminate GBM cells through reactive oxygen species-mediated apoptosis [155]. Liang et al. (2021) showed the theranostic application of a poly(2-methoxy-5-(2′-ethylhexyloxy)-p-phenylenevinylene) NP modified with anti-EGFRvIII, which effectively targeted GBM cells, enabling both imaging of the tumor and PDT [156]. Poly(9,9-dioctylfluorene-alt-benzothiadiazole) and poly(styrene-co-maleic anhydride) NPs doped with platinum porphyrin, combined with metronomic PDT (i.e., lower irradiation rates delivered over a longer time), have also been confirmed to be efficient in GBM therapy [157].

Multifunctional NPs encapsulating lexiscan (i.e., Regadenoson; a BBB modulator) have been found to effectively cross the BBB via autocatalytic delivery [158]. This two-step approach is beneficial as, firstly, NPs carrying lexiscan pass through the BBB either via transcytosis or gaps in the barrier, which is then followed by release of the BBB modulators within the TME. This results in a transient increase in the permeability of the BBB [158]. Wu et al. (2022) found that the conjugation of a lexiscan-loaded PEG–glutathione-reactive poly (2,2″-thiodiethylene 3,3″-dithiodipropionate) polymer with iRGD (a cyclic peptide; increases BBB crossing) and modification with neutrophil elastase (enables shrinking of the nanocomplex), allowed the effective passage of the BBB. Additionally, loading the nanocarrier with DOX and chlorin e6 (a sonosensitizer) prolonged the survival of GL261 glioma-bearing mice by 20 days in comparison to mice treated with free-DOX or DOX/chlorin e6, with no observed adverse effects [159]. Another method of alleviating the passage of CPNs across the BBB is the usage of monocytes as carriers of these nanoparticles [152]. Finally, passage of the BBB can be achieved using a diketopyrrolopyrrole-based conjugated polymer with fluorescein-conjugated hyaluronic acid, which can target tumor-initiating cells that express CD44 (correlated with GBM progression) [151].

### 5.4. Polymeric Micelles

Micelles are a prominently researched area of polymeric NPs that, comparably to the aforementioned NPs, provide a holistic approach to GBM therapy. These molecules may arrange themselves in a spherical form in aqueous solutions, encapsulating substances inside them. The micelle particle diameter ranges from 5 to 100 nm. They may efficaciously transport chemotherapeutics (e.g., DOX and its derivatives), cytostatics (e.g., imatinib or panobinostat), photosensitizers for PDT, as well as statins (i.e., pitavastatin) [160,161,162,163,164]. Micelles have been found to be a safe and effective method of inducing an anti-glioma effect and prolonging the survival of xenograft rats or mice via various routes of administration, including intranasal, oral, and intravenous [165,166,167]. An important advantage of micelles is their ability to cross the BBB and enhance the potential of antitumor therapy [168], including through targeting proteins (e.g., glucose transporter 1) often overexpressed in cancer cells [169]. Micelles are also modified using specific peptides to enable αv β integrin and NRP1-mediated ligand transfer across the BBB [168]. Therefore, the combination of micelles’ ability to penetrate the BBB and specifically target tumors is a promising step forward in cancer therapy. 

#### 5.4.1. Improvement of Chemotherapeutic Efficacy in Micelle-Based Approaches

To increase the outcomes from the treatment of GBM using chemotherapy, various combinatory modalities are being explored, including the conjugation of carmustine-loaded micelles with the Pep-1 cell penetrating peptide and borneol, which markedly suppressed the growth of gliomas and prolonged the survival of the tested Luc-BT325 mice, while presenting limited side effects [170]. The improvement of the anticancer action of micellar strategies, through targeting and limiting the viability of GSCs, has also been attained by developing polymer–micellar NPs containing both TMZ and idasanutlin (an inhibitor of the mouse double minute 2 homolog) [171]. Apart from the restriction of the proliferation of cells and increased apoptosis, an anticancer effect was attained via the inhibition of angiogenesis using poly(styrene-co-maleic) acid micelles carrying crizotinib and dasatinib [172]. 

#### 5.4.2. Micelles as Nucleic Acid Transporters

Similarly to the previously described NPs, micelles are being considered for the effective transport of siRNA to tumor cells, such as the thermoassemble ionizable reverse pluronic system devised by De et al. (2024), which assumed the form of a micelle and could carry siRNA targeting Bcl2 (apoptosis regulator) across the BBB [173]. Moreover, micelles dual loaded with curcumin and an antisense oligonucleotide against miR-21, which is commonly overexpressed by GBM cells, have been found to effectively lower the level of miRs upregulated in gliomas [174]. Aside from siRNA and miRs, micelles have also been modified by: (i) the introduction of a pH-sensitive masking sequence, allowing for targeted drug release; or (ii) the construction of pH and glutathione dual-responsive copolymers containing polo-like kinase 1 (PLK1)-specific short-hairpin RNA and DOX [175,176]. Furthermore, the usage of micelles in combination strategies for carrying drugs and siRNA has been shown to be effective in inhibiting proliferation and initiating apoptosis of glioma cells, as well as prolonging the survival of xenograft GBM mice. Such results were obtained for both folate-targeted micelles loaded with TMZ and anti-BCL-2 siRNA, and micelles carrying TMZ and siRNA targeting polo-like kinase 1 (modified with Ang-2) [177,178].

#### 5.4.3. Micelles Combined with Phototherapy, Radiotherapy, or Immunotherapy

Aside from chemotherapy, micelles display beneficial results for the enhancement of phototherapy, alongside increased drug delivery, in the treatment of GBM [179]. The application of micelles in photoacoustic imaging is promising, as they demonstrate exceptional photostability and are effective at photothermal conversion [180]. They have also recently been reported to improve radiotherapy, due to their ability to transport radiosensitizers [181]. After treating U251 GBM-bearing mice with micelles carrying both the radiosensitizer Dbait and DOX, their survival was reported to be elongated by 30 days, relative to the control [182]. 

Finally, the usage of micelles in immunotherapy deserves attention, as pH-sensitive epirubicin-loaded micellar complexes combined with anti-PD1 antibodies have been found to effectively eliminate GBM resistance in vitro [183]. Micelles co-loaded with PTX and anti-programmed death-ligand 1 antibodies have also been observed to induce immune memory in GBM-carrying mice, while maintaining good stability [184].

#### 5.4.4. Theranostic Application of Micelles

Alongside the auspicious role of micelles for drug delivery, they are also a targeting moiety for molecular markers overexpressed in glioma cells; therefore, they can be significant for both imaging and therapy [185]. Antitumor glycoside-coated micellar iron oxide NPs may be potent MRI contrast agents, while retaining their antitumor abilities [186]. The theranostic application of micelles for chemotherapy and MRI imaging was also discussed using the example of oleic acid-modified manganese oxide- and TMZ-loaded polymeric micelles, encompassing the internalization of arginine–glycine–aspartic acid. Efficient penetration of the BBB allowed for the accumulation of the nanocarrier and the release of TMZ, Mn^2+^, and O_2−_ at the tumor site, which in turn initiated cellular apoptosis and death of the tumor cells [187]. These findings were confirmed using modified polyprodrug amphiphiles, which exhibited comparable qualities [188].

#### 5.4.5. Novel Approaches to Micelles

The investigation of “smart” multifunctional nanoparticles and dual-targeted drug release vehicles is becoming more common [189]. The main advantages of such novel systems, such as those involving super-small zwitterionic micelles [190], intrinsically disordered protein micelles [191], or docetaxel-carrying ultra-small micelles [192], include more efficient bypassing of the BBB due to their reduced size, resulting in improved delivery of chemotherapeutics to the target cells and, thus, longer survival of orthotopic GBM mice. Such smart nanovehicles can also increase the efficacy of molecularly targeted treatment strategies, e.g., those involving sorafenib [193]. Another promising alternative to micelles may be micelleplexes, characterized by the presence of transported nucleic acids in the corona of the polymer micelle. Short nanofiber micelleplexes have especially been indicated as promising transporters, due to their higher cargo loading properties and better transfection of GBM cells [194].

Furthermore, modifications in the architecture of micelles, such as the formation of star-shaped polymers [195,196] or 3-helix micelles [197], may become an important factor in micellar therapies in the future, due to the enhanced capacity of the NPs and improved kinetic properties. Other alterations to micelles include the formation of multipurpose cation-free siRNA micelles, which effectively circumvent the BBB via receptor-mediated transcytosis, that can be loaded effectively with chemotherapeutics (e.g., TMZ), and are efficient at distributing drugs at the GBM site [198].

Finally, apart from structural innovations, improvements in the delivery of micelles to GBM cells are being sought. A promising proposal that has also been used in liposomal approaches (discussed above) is the facilitation of the distribution of these NPs through the use of microbubble-based sonoporation, as this method enables the homogenization of micellar transport to target tissues and elongates the residence of micelles at the tumor site [199].

### 5.5. Dendrimers

Dendrimers are branched organic molecules, composed of an inner and outer shell encircling a linear or small molecule core. Dendrimers were first assessed as drug-delivery systems in 1982 and, since then, there has been constant interest in their application in cancer therapy [200]. Similar to other NPs, their targeting and distributive abilities are dependent on the size of the nanovehicle [201]. Their main advantages include stability, solubility, limited side effects, and the ability to self-assemble, which may be implemented in the bioimaging of tumors [202,203]. They are often applied for the treatment of CNS disorders, due to their anti-inflammatory abilities [204]. 

Polyamidoamine (PAMAM) dendrimers are the most prevalent class of dendrimers. They are characterized by an ethylenediamine core and are often applied in biotechnological studies. In GBM therapy, PAMAM dendrimers have been conjugated with various anticancer agents, including celecoxib (a cyclooxygenase-2 inhibitor), simvastatin (a HMG-CoA reductase inhibitor), curcumin, or etoposide (a standard chemotherapeutic drug that disrupts topoisomerase II action) [205], leading to enhanced drug properties, as well as exhibiting a synergistic effect when combined with phototherapy [206,207,208]. The conjugation of PAMAM dendrimers with sugar (glucose, mannose, and galactose) moieties has also led to improved specificity of GBM cell targeting [209]. Uram et al. (2019) reported increased apoptosis, and decreased proliferation and migration of U-118 GBM cells treated with biotinylated PAMAM G3 dendrimers conjugated with celecoxib and peroxisome proliferator-activated receptor γ (against Fmoc-L-Leucine) [210]. A reduction in the proliferation of U-118 glioma cells and an improvement in drug toxicity were also achieved through the combination of dendrimers with α-mangostin (a polyphenolic xanthonoid) and vadimezan (a flavone-acetic acid-based drug) [211]. Furthermore, PAMAM dendrimers have been applied for the encapsulation of both arsenic trioxide and TMZ for improved penetration of the BBB, an enhanced antitumor effect, and more effective delivery of the drug to GBM cells [212,213]. A spinoff from this study revealed that the internalization of the RGD recognition ligand of the integrin αvβ3 receptor and the BBB-targeting group TGN allows for improved therapeutic outcomes, while restricting the side effects of arsenic trioxide [214]. 

Liu et al. (2019) reported that Ang-2-functionalized PAMAM dendrimers present high BBB permeability and may be further functionalized with the EGFR-targeting peptide for enhanced tumor-targeting efficacy both in vitro and in vivo [215]. Han et al. (2018) similarly showed that Ang-2-modified DOX-loaded PAMAM dendrimers present increased affinity to the target cells and improve cellular uptake. The antitumor efficacy of the modified dendrimers was further improved after combination with borneol [216]. Further confirmation of the beneficial properties of Ang-2 PAMAM dendrimers was confirmed by Sahoo et al. (2023)*,* who found that the encapsulation of TMZ and the PEGylation of NPs results in ~250 nm particles that present improved cellular uptake in U-87 cells, an over 2-fold increase in the half-life, and a >3-fold improvement in brain uptake in the rat model, when compared to free TMZ [217].

The conjugation of dendrimers with cytotoxic and cell-penetrating peptides, as shown by Liu et al. (2018), has achieved promising results, including enhanced tumor penetration, improved intracellular peptide delivery, and increased antitumor effect. Interestingly, the anticancer effect of the devised nanosystem was achieved through the deprotection of MMP-2-PEG, which exposed the cytotoxic and cell-penetrating peptides and allowed them to enter the tumor cells [218]. Wu et al. (2018) found that the conjugation of the mesenchymal–epithelial transition factor-targeting cMBP peptides and G4 dendrimers inhibits the growth of glioma, hindering downstream signaling, and causing a decrease in the proliferation of U-87 GBM cells [219].

Moreover, the role of amphiphilic dendrimers in the delivery of siRNA to microglia may be key for developing strategies based on the knockdown of strategic genes regulating the carcinogenic process [220]. Jin et al. (2021) assessed modified PAMAM dendrimers loaded with siRNA (siLSINCT5), decorated with cell-penetrating peptides and, subsequently, conjugated with aNKG2A (a checkpoint inhibitor), for the efficient delivery of nucleic acid through the BBB. They confirmed that the nanosystem not only presented favorable BBB-passing properties, but also elongated the survival of U-87 xenograft mice by >20 days, in comparison to those treated with dendrimers containing only siLSINCT5 [221].

Sharma et al. (2020) and Liaw et al. (2021) broadly explored the potential application of dendrimers in immunotherapy of GBM, specifically as vehicles for targeting tumor-associated macrophages (TAMs). They found that dendrimer–rapamycin conjugates were precisely localized in TAMs and, additionally, the antiproliferative action of rapamycin was enhanced, leading to a decrease in the tumor burden [222]. Importantly, due to the ability of dendrimers to target TAMs in GBM, after specific modifications, they were proposed as potential NPs for the organelle-targeted delivery of chemotherapeutics [222]. Interestingly, they also reported that triptolide (a diterpenoid epoxide) presented decreased adverse effects, while sustaining its antiproliferative and immunosuppressive effects, which was attained through specific targeting of TAMs through the formation of conjugated dendrimer–triptolide structures [223]. 

Future development of dendrimers may include the construction of telodendrimer nanocompositions, characterized by a small size and effective drug-delivery abilities [224]; nanoantidotes that encompass cysteine-wrapped dendrimers and that transport TMZ [225]; or nanodiamonds functionalized with PAMAM dendrimers for more efficient delivery of cargo to target GBM cells [226]. Also, the construction of biopolymers containing dendrimers may be used for the encrustation of a DOX-loaded PLGA nanoparticle core, thus improving the delivery of chemotherapeutics across the BBB and the subsequent antitumor action [227]. Lastly, a novel method of increasing the effectiveness of dendrimer-based anti-GBM therapies is the usage of combinatory strategies involving PEGylated graphene oxide-carrying CPI444 and vatalanib (anticancer compounds). This allows for the limitation of migration and the increased apoptosis of U-87 GBM cells [228].

### 5.6. Nanogels

Recent years have brought significant advancements in the field of nanogels. They are a combination of nanoscale technology with hydrogels, possessing the characteristics of both. Their exceptional biocompatibility and biodegradability, supported by low toxicity (especially when compared to other NPs), has attracted the attention of the scientific community. Nanogels are hydrophilic, three-dimensional structures, with high incorporation capacity, exceeding 30%. In addition, their specification and properties, such as softness, porosity, size, amphiphilicity, and degradability, can be modified through alterations in their formulation. Different stimuli, including alterations in the pH value, temperature, redox potential, UV light, or magnetic field, might lead to changes in the conformation of nanogels, influencing their hydrophilic/hydrophobic properties [229].

#### 5.6.1. Locally Delivered Nanogels

Due to their biological properties and low cytotoxicity, hydrogels can be delivered and remain in the body for the gradual release of drugs. Moreover, they can adapt their shape to the surrounding tissues [230]. Different nanogels have also been used to locally deliver various drugs with limited ability to pass through the BBB [231,232]. The natural location for nanogel insertion is the niche created after tumor resection. A nanogel consisting of PEG and PLGA (PEG-PLGA), co-loaded with curcumin and TMZ, injected into the postsurgical cave, significantly reduced relapse due to residual GBM cells [233].

An interesting system was produced by Godau et al. (2023), who managed to develop a shear-thinning hydrogel, where the gel thinned under pressure, allowing the controlled release of DOX. The results showed that this gel could effectively release DOX over long periods of time (up to 80 days), thus reducing the side effects. When injected into tumor tissue, it improved survival rates in GBM models by 50% for 22 days and 25% for 52 days [234]. Nanogels also possess the ability to locally deliver drugs under changing conditions, such as the pH, redox, or magnetic field [235,236]. 

Therapies for GBM treatment utilizing non-coding RNAs or miRs have also been studied. Shatsberg et al. (2016) constructed nanogel–miR-34a nano-polyplexes that mask the negative charge of miRNA and transport it to the cytoplasm of GBM cells. In vivo studies have shown that the miR-34a-armed nanogel reduced tumor growth in comparison to the control [237]. PLGA-based nanogels have also been employed to deliver dextran (a ligand binding to the translocator protein), which is a mitochondrial target for anticancer therapy [238]. Gao et al. (2021) developed a nucleic acid nanogel coated with a membrane mimicking that of a virus (Vir-Gel) loaded with miRNA. Vir-Gel imitated a viral infection increasing nanogel uptake and prolonged blood circulation, while miR-155 inhibited GBM growth [239].

Nanogels might also be used to provide therapy that is unavailable in natural conditions. An example is PDT, which is a promising anticancer treatment with reduced side effects; however, due to hypoxia of the TME and the highly hydrophobic properties of photosensitizers, such use is significantly limited. Nevertheless, a H-DNA self-assembly nanogel can deliver oxygen and photosensitizers required for therapy [240].

#### 5.6.2. Nanogels Crossing the Blood–Brain Barrier

The construction of BBB-crossing nanogels is challenging. In order to address that issue, Li et al. (2023) coated DOX-loaded nanoparticles with platelet membranes. The tool allowed BBB penetration, prolonged the survival of GBM-bearing mice, and limited the immune response [241]. On the other hand, Zhang et al. (2022) utilized an erythrocyte membrane enriched with the ApoE peptide [242]. Another approach involved a pH/redox-sensitive carboxymethylchitosan nanogel fortified with the peptide Ang-2, which enhanced BBB passage and targeted the cancer cells [145]. Thermosensitive nanogels armed with cell-penetrating peptides provide a promising drug-delivery platform that is able to actively penetrate the BBB. Furthermore, nanogel compounds passively target spots with elevated temperature, like the TME, allowing for the local delivery of therapeutic agents [243]. Enriching nanogels with the metalloproteinase 2/9 substrate increases the transcytosis of NPs via the BBB and allows further release at the GBM site [244]. Alternatively, the modification of nanogel particles with lactoferrin and phenylboronic acid or antibodies is yet another way to increase BBB permeability [245,246,247]. 

Nanoparticles that do not require advanced modifications to overcome the BBB are of particular interest. She et al. (2020) synthesized a hypoxia-activated drug-loaded nanogel that presented high biocompatibility and could last a long time in the bloodstream. The nanogel effectively crossed the BBB, due to the phosphorylcholine-enriched formulation that mimicked the structure of the cell membrane [248]. 

Nanogels may be implanted during surgery; however, there are studies on non-invasive intranasal nanogel delivery [249]. Gadhave et al. (2021) implemented a new nano-lipid-based carbopol–gellan gum, in situ gel, loaded with teriflunomide (TNLCGHG). The nanogel exhibited mucoadhesive properties and in vivo studies on mice confirmed its nasal permeation [249]. 

Nanogels delivered in situ enable the direct administration of therapeutic agents, limiting their side effects. In addition, this strategy allows immune response escape. However, it requires surgical resection, which is not always possible, like in non-surgical GBM patient cases. In such situations, nanogels capable of crossing the BBB are an option. Nevertheless, these therapies are more laden with side effects and usually require higher doses of the drug and/or increased frequency of administration. It seems appealing to consider a transdermal drug-delivery method that can combine the advantages of the previous approaches, while reducing adverse effects.

## 6. Carbon Nanotubes

Carbon nanotubes (CNTs), which are composed of carbon molecules organized in the form of graphene, are divided into two subtypes, single-wall or multi-wall CNTs, based on the number of layers of graphene [250,251]. The advantages of single-wall carbon nanotubes (SWCNTs) include a simple structure and increased flexibility, while multi-wall CNTs (MWCNTs) are characterized by higher accumulation in tissues and greater purity [252]. Also, due to their ability to convert near-infrared light into heat and, thus, heat cancer cells, CNTs may in the future be applied in hyperthermia treatment of GBMs [253]. 

To improve the efficacy of SWCNTs, especially in regards to their accumulation and distribution in glioma cells, the exposure to a low-strength electric field has been considered [254]. Another method of improving the properties of SWCNTs is modification through the addition of various chemical groups, which broaden the spectrum of features of NPs and improve the capacity of the drugs that reach the tumor [255]. Water-soluble SWCNTs, functionalized with either PEG or tetrahydrofurfuryl-terminated PEG, were found to induce alterations in the morphology of D54 glioma cells, reduce their proliferation, and increase the rate of cell death [256]. The functionalization of SWCNTs has also been achieved using CpG-oligodeoxynucleotides, which stimulate toll-like receptor-9 and activate the immune system [257]. CpG-oligodeoxynucleotides were previously found to inhibit the development of tumors, including gliomas in the xenograft model, although the efficacy in patients is limited. Thus, it was considered that the functionalization of SWCNTs with CpG may improve the clinical effectiveness of CpG in humans [257]. Remarkably, the SWCNTs/CpG construct concurrently inhibited the migration of glioma cells and activated the immune system [258].

Regarding multi-wall CNTs, nanosystems consisting of hydroxylated MWCNTs and all-trans retinoic acid, which were loaded on electrospun polycaprolactone nanofibers, have been reported to effectively reduce the viability and stemness of GSCs and may, therefore, be promising nanoplatforms for enhancing currently available treatment strategies via concurrent heat and differentiation therapy [259]. 

In parallel with other N-M NPs, carbon nanotubes are also being combined with metal particles, such as the metal CNTs devised by Wang et al. (2023). They reported that such iron-filled CNTs with a carbon outer layer, which were functionalized with the anti-CD44 antibody, enabled the magnetic field treatment of chemoresistant GBMs. The administration of a series of magnetic treatments resulted in an over 2-fold reduction in the tumor area, relative to the control mice [260]. 

A novel approach to CNTs is the application of carbon nanotube sponges (CNSPs) doped with nitrogen. CNSPs present favorable drug-carrying properties and may be loaded with chemotherapeutic agents that have a total weight equal to 100-fold that of the CNSPs alone. Nevertheless, initial in vitro studies conducted on CNSPs loaded with carmustine (a chemotherapeutic) have indicated that the difference in cytotoxicity between drug-carrying CNSPs and carmustine administered alone was not significant [261].

Nevertheless, the potential side effects of the treatment of patients with CNTs require careful consideration. Despite the CNTs’ exceptional properties for cell scaffolding, loose CNTs have been reported to instigate toxicity in vivo, while in vitro studies have shown that the extended growth of U-87 GBM cells on CNT-coated scaffolds promotes their proliferation [262]. SWCNTs have also been reported to dysregulate the expression of certain regulatory genes (including *DNAJB9*, *TOB1*, *BRCA1*, and *P4HA2*) more strongly in normal astrocytes (the NHA/TS cell line) than U-87 GBM cells [263]. Therefore, it is essential to better understand these NPs before administrating them to cancer patients.

## 7. Silica and Selenium-Based NPs

Interest in silica nanoparticles (SiNPs) as potential treatment modalities for gliomas has increased over the past 10 years. SiNPs are recognized as biocompatible and multifunctional vehicles, with applications in the targeted delivery of drugs (e.g., TMZ), methotrexate (an immunosuppressant), or tyrosine kinase inhibitors, such as ponatinib (an abnormal tyrosine kinase inhibitor targeting BCR-ABL) [264,265,266]. Their size ranges from 2 to 50 nm. Initially, the anticancer properties of amorphous silicon dioxide (SiO_2_) NPs were explored; however, it was found that SiO_2_ itself presents toxicity and therefore raises some safety concerns, even though small SiO_2_ NPs (~15 nm) have mainly been considered as toxic, rather than larger ones. Various studies have reported a significant dependence of the anticancer properties of SiNPs on the dose, as well as the type of cancer cells used in the tests. This suggests that the exposure conditions of SiNPs, including variations in their size, can likely affect different cellular pathways of GBM cells [267,268].

Several clinical trials on the application of SiNPs (including mesoporous silica NPs and Cornell dots) have been conducted, which have confirmed their safety and efficacy in humans after oral administration, as well as their promising role as imaging probes; however, these studies did not involve the treatment of GBM patients [269,270]. Moreover, despite the abovementioned advantageous features of SiNPs, several issues have to be resolved, including the determination of their safety after a long exposure time, enhancing their encapsulation effectiveness, and investigating their effect after intravenous administration [270].

### 7.1. Silica Nanoparticles

#### 7.1.1. Mesoporous Silica Nanoparticles

Mesoporous silica nanoparticles (MSNPs) present a porous nature and, therefore, have been proposed as a promising novel alternative to the traditional methods of amorphous formulation development, as they offer not only high pore volumes and surface areas, but also biocompatibility [271]. The large surface area of MSNPs possesses a high content of silanol groups (Si-OH), which can be manipulated as the site of attachment for surface probes. Apart from a high-loading capacity, they offer improved passage of chemotherapeutics across the BBB and aid the localization of drugs in malignant cells [139,272,273]. MSNPs loaded with prodrugs have also been confirmed to enhance tumor suppression. An interesting strategy involved thymoquinone (a natural prodrug)-loaded MSNPs, which were encapsulated in a shell of whey protein and gum Arabic. This complex presented augmented release in an acidic pH and an increased cytotoxic effect on glioma cells, and especially enhanced apoptosis and G2/M phase cell cycle arrest [274]. Apart from classical chemotherapeutics, MSNPs loaded with lactate (an anti-GBM factor) have proven to increase plasma acidification and, thus, induce cytotoxicity of C6 cells (a glial cell line isolated from the brain of a rat with glioma) in vitro and prolong the median survival of malignant glioma-bearing rats by 35 days in vivo [275].

#### 7.1.2. Novel Approaches to Mesoporous Silica Nanoparticles

MSNPs can be further functionalized to enhance their drug-loading capacity through the construction of nanoplatforms. An example of such an attempt are Ang-2-modified lipid-coated MSNPs loaded with PTX (ANG-LP-MSN-PTX). It was reported that such a biocomplex increases the transport of PTX over the BBB through the enhanced targeting of low-density lipoprotein receptor-related protein 1 (LRP1), which plays an essential role in cell signaling. Also, the observed improved targeting effectiveness of the chemotherapeutics resulted in the prolonged survival of the tested rats by ~10 days relative to the rats treated with PTX alone [276]. Comparable results for Ang-2-modified liposome–silica hybrid NPs with attached polyacrylic acid were obtained when the nanoplatform was applied for the delivery of arsenic trioxide to glioma cells [277]. Furthermore, integrin-targeted ultrasmall fluorescent core–shell SiNPs with attached kinase inhibitors (such as dasatinib, a dual Src/Abl kinase inhibitor) were proposed by Juthani et al. (2020) to enhance drug delivery into GBMs [278]. 

An interesting approach proposed by Sapre et al. (2019) considered the administration of silica-cloaked adenoviruses or virus-like silica NPs (VSNPs), as they possess beneficial properties, i.e., a spiky surface, for the penetration of the BBB. The adenovirus SiNPs were reported to improve the cargo delivery and did not induce immunogenicity. The following studies on the effect of rough-surface VSNPs on the integrity and permeability of the BBB in vitro showed a greater effect on the transient opening of endothelial tight junctions of the BBB, when compared to smooth-surface Stöber SiNPs [279,280]. It was proposed that the rough surface of VSNPs increases their adhesion to cells building the BBB and also offers a larger surface area for interacting with BBB-interfering proteins [280]. Furthermore, Zhang et al. (2019) proposed the concurrent treatment of cells using drug-loaded MSNPs and inhibitors of autophagy. Polydopamine-coated and TMZ-loaded MSNPs, conjugated with Asn-Gly-Arg (NGR), were found to increase the autophagy and apoptosis of C6 glioma cells, leading to an improved anti-GBM effect [281]. 

Despite the numerous advantages of enriching MSNPs with targeting moieties, several drawbacks are recognized, which include problems with circulation and rapid clearance of the MSNPs from the blood [282]. A promising alternative is receptor ligand-free MSNPs. Chen et al. (2024) reported that ligand-free PEGylated MSNPs characterized by a small size (25 nm; better delivery across the BBB) and near-neutral charge presented an increased blood circulation time (>24 h). Also, when the MSNPs were loaded with DOX, the accumulation of the drug in the brain of U87-LUC orthotopic xenograft tumor BALB/c nude mice was enhanced 6-fold, relative to the free drug [282]. 

#### 7.1.3. Theranostic Application of Mesoporous Silica Nanoparticles

Interest in the theranostic application of MSNPs has increased recently, as nanoplatforms have been considered in both GBM therapy and image-guided tumor localization. Frequently, the nature of such nanoplatforms is multicomposed. In general, the main expectations from such a tool are to achieve immune response tolerance, as well as an improvement in drug/gene therapy, PDT, and photothermal therapy. Li et al. (2022) published data on the anti-GBM properties of MSNPs containing indocyanine green dye (presents photothermal properties), miR (as a therapeutic agent; miR-137), and RGD-modified (Arg-Gly-Asp-d-Phe-Cys peptide selectively targeting tumor integrins) red-blood-cell membranes (to secure long circulation and immune escape) [283]. Another specially constructed Si-based NP, composed of an iron oxide core within a mesoporous silica shell (Fe@MSNs), delivered with 1400 W (a specific inhibitor of inducible nitric oxide synthase) to brain tumor-initiating cells was also shown to be capable of suppressing tumor growth and increasing the survival of GBM-bearing mice. The release of the 1400 W compound was attained through the application of an external low-power radiofrequency field [284]. For application in MRI tracking, DOX-loaded magnetic MSNPs have been internalized in inflammation-activatable neutrophils. The treatment effectively increased the intratumoral accumulation of the drug, without impacting the neutrophils’ viability. This method may delay recurrence of the glioma, thus extending the survival of patients [285].

Regarding future application of MSNPs in GBM treatment, polyethyleneimine-functionalized MSNPs, which can specifically target GSCs without affecting normal brain cells, are an interesting approach [286]. Moreover, hybrid metal SiNPs (e.g., silver–silica NPs) may also be promising for theranostics, as such hybrid NPs offer promising biocompatibility and an anti-GBM effect, while also presenting fluorescence for diagnostic purposes [287].

### 7.2. Selenium Nanoparticles

Selenium nanoparticles (SeNPs) have recently been conceded as effective anticancer molecules, as several research groups have reported on their pro-apoptotic properties. It was shown that SeNPs are capable of inducing apoptosis of glioma cells in vitro, including both drug-sensitive and drug-resistant cells (A172 and T98G, respectively) [288,289,290,291,292]. Also, a nearly 2-fold increase in apoptosis and death of glioma cells due to exposure to TMZ-loaded selenium NPs coated with methacrylic acid (Eudragit) and chitosan was reported. This phenomenon was accompanied by a significant decrease in the half-maximal inhibitory concentration (IC_50_) level and a reduction in the expression of TMZ resistance-related genes (including *MGMT*, *E2F6*, and *RELA*) [293]. 

SeNPs have also been used for the creation of nanocomplexes. An example of such vehicles are SeNPs containing sorafenib, an inhibitor that blocks angiogenesis and tumor cell proliferation through the inhibition of kinases, such as VEGFR2, PDGFR, or the serine/threonine kinase RAF [294]. It was found that the antitumor effect of the selenium–sorafenib nanocomplex is related to the altered expression of selenoproteins through the Ca^2+^-dependent induction of endoplasmic reticulum stress and kinase regulators of oncogenicity.

Importantly, SeNPs may act as a theranostic tool, aiding the navigation of the complex BBB and supporting targeted delivery of chemotherapeutics [295]. Furthermore, promising results were obtained for the devised nanoplatform consisting of HER2 antibody-conjugated SeNPs. MRI revealed the successful delivery of SPIONs to the brain via the novel system, indicative of HER2@NPs disrupting the integrity of the BBB [295]. Selenium-containing nanotools have also been proposed as radiosensitizers. Tang et al. (2023) showed that biodegradable selenium-engineered mesoporous silica nanocapsules carrying siRNA targeting cofilin-1 (a protein connected to tumor progression), and triggered by X-ray irradiation, enhanced the survival of U-87-Luc orthotopic GBM mice (relative to the non-treated group) by >2-fold [296].

## 8. Conclusions

Despite constant progress in cancer therapy, glioblastoma still remains a cancer characterized by low survival rates and a lack of effective therapeutic methods. Therefore, there is a need to improve existing treatment approaches and develop novel therapeutic schemes to treat GBM and significantly improve patient survival. A promising anticancer strategy, which is an alternative to conventional therapeutic methods and allows targeted drug delivery, involves the use of nanoparticles.

**Figure 3:**
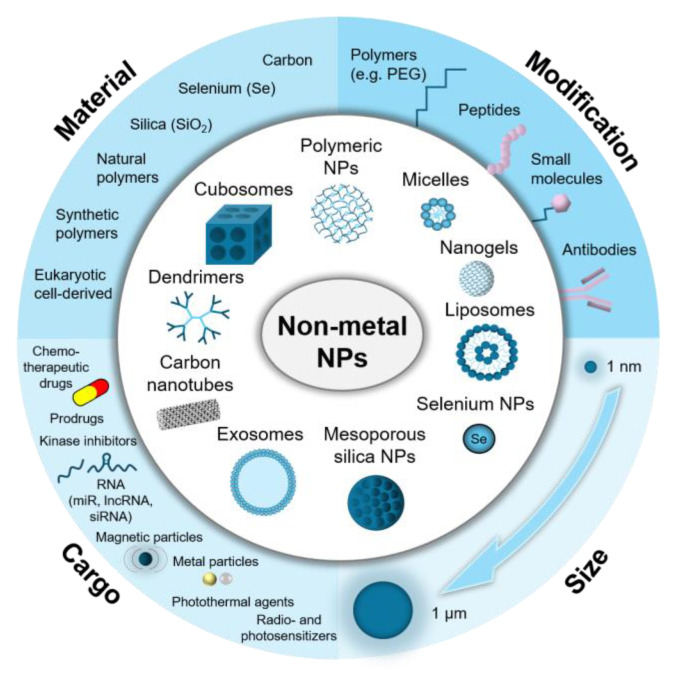
Schematic representation of the main non-metal nanoparticle-based drug-delivery systems discussed in this review and their characteristics: material of origin, functional surface modification, particle size, and cargo (modified from [298]).

NP-based technologies offer a variety of solutions. Among the promising non-metal based nanotechnological strategies in the treatment of GBM, the most commonly investigated approaches are based on the application of exosomes, liposomes, hydrogels, and micelles as: (i) targeted drug-delivery systems (delivery of a varied palette of chemotherapeutics, including TMZ, DOX, and PTX) or (ii) nanovehicles for the transfer of nucleic acids (i.e., siRNA or miRNA). Additional approaches involving cubosomes, carbon nanotubes, dendrimers, and Si- or SeNPs, which often offer better drug-loading and targeted distribution properties, alongside sufficient stability and the ability to overcome the BBB, are also studied (Figure 3). 

Nanoparticle use in drug delivery offers a variety of advantages that further prove their therapeutic potential. They can carry multiple molecules due to their high-cargo capacity and possess the ability to bind both hydrophobic and hydrophilic compounds. Nanoparticles also enable increased targeting of cancer cells when decorated with specific ligands, as well as other modifications to increase the accessibility and cytotoxic properties of drugs. Currently, the new reports on targeted NPs (loaded with a CSP prodrug) show their ability to effectively target both peripheral and brain tumors, as well as successfully overcome the BBB [297]. Other significant advantages of NPs include their low toxicity, thermodynamic stability, and sustained drug release. Overall, they are a promising platform to enhance treatment efficacy, especially since some of these approaches have already shown promise in vivo or even in clinical trials.

Nevertheless, apart from their numerous advantageous features, certain obstacles in the application of these NPs are still present and require further study. Studies on nanocarriers show the complexity of the current challenges in NP research, which include the complexity of the synthesis of NPs, the presence of radio- and chemoresistant GSCs, limited transport across the BBB, or insufficient delivery of cargo to the tumor site. Conventional drug-loaded nanovehicles may leak or readily release the compounds, so specific designs for NPs carrying active compounds are required to assure their full effectiveness. Moreover, the uptake of nanoparticles by GBM cells and, therefore, their effectiveness, may be restricted by external factors, such as heparin. An important issue is also the administration of NPs to patients and their further passage through human organs, especially the liver. This issue is particularly relevant for lipid nanocarriers, which may be consumed by the liver, preventing therapeutic concentrations from being reached. Nonspecific uptake of drug-loaded NPs by body tissues can cause damage, which could exclude or significantly limit the use of such therapies. The solution may be to enrich the carriers with targeting components; however, the challenge lies in finding such moieties that target only GBM cells and do not interfere with BBB penetration. One solution to this problem involves local administration of the drug into the postoperative niche, but it is not always possible to provide immediate therapy and not all tumors are operable.

Overall, in spite of their limitations, the discussed non-metal nanoparticle-based strategies carry great promise, as they offer a variety of therapeutic approaches, especially in the field of targeted drug delivery. Extensive research and focus on developing novel and refining current NP-based formulations, as well as their use in nanomedicine, may enhance their translation into clinical settings and, ultimately, contribute to overcoming the challenges of cancer treatment.

## Abbreviations

Ang-2	angiopep-2
ApoE	apolipoprotein E
BBB	blood–brain barrier
CNS	central nervous system
CNSP	carbon nanotube sponge
CNT	carbon nanotube
CPNs	conjugated polymer nanoparticles
CSP	cisplatin
DOX	doxorubicin
EGFR	epidermal growth factor receptor
GBM	glioblastoma multiforme
FDA	Food and Drug Administration
GSC	glioma stem cell
lncRNA	long non-coding RNAs
MGMT	O^6^-Methylguanine-DNA methyltransferase
MiRNA; miR	microRNA
MMP-2	matrix metalloproteinase-2
MRI	magnetic resonance imaging
MSC	mesenchymal stem cell
MSNP	mesoporous silica nanoparticle
MWCNT	multi-wall carbon nanotube
N-M NP	non-metal-based nanoparticle
NP	nanoparticle
NRP1	neuropilin-1
PAMAM	polyamidoamine
PBAE	poly(β-amino ester)
PCL	poly-ε-caprolactone
PDT	photodynamic therapy
PEG	polyethylene glycol
PFS	progression-free survival
PLGA	poly(lactic-co-glycolic acid)
PTX	paclitaxel
SeNP	selenium nanoparticle
SiNP	silica nanoparticle
SiO_2_	silicon dioxide
siRNA	small interfering RNA
SPION	superparamagnetic iron oxide nanoparticle
SWCNT	single-wall carbon nanotube
TAM	tumor-associated macrophage
TME	tumor microenvironment
TMZ	temozolomide
VEGF	vascular endothelial growth factor
VSNP	virus-like silica nanoparticle

## References

[1] Kanderi T., Munakomi S., Gupta V. (2024). Glioblastoma Multiforme. StatPearls.

[2] Ostrom Q.T., Rubin J.B., Lathia J.D., Berens M.E., Barnholtz-Sloan J.S. (2018). Females have the survival advantage in glioblastoma. Neuro Oncol..

[3] Colopi A., Fuda S., Santi S., Onorato A., Cesarini V., Salvati M., Balistreri C.R., Dolci S., Guida E. (2023). Impact of age and gender on glioblastoma onset, progression, and management. Mech. Ageing Dev..

[4] Efremov L., Abera S.F., Bedir A., Vordermark D., Medenwald D. (2021). Patterns of glioblastoma treatment and survival over a 16-years period: Pooled data from the German Cancer Registries. J. Cancer Res. Clin. Oncol..

[5] Sweeney M.D., Zhao Z., Montagne A., Nelson A.R., Zlokovic B.V. (2019). Blood-Brain Barrier: From Physiology to Disease and Back. Physiol. Rev..

[6] Lombardo S.M., Schneider M., Tureli A.E., Gunday Tureli N. (2020). Key for crossing the BBB with nanoparticles: The rational design. Beilstein J. Nanotechnol..

[7] Gawel A.M., Singh R., Debinski W. (2022). Metal-Based Nanostructured Therapeutic Strategies for Glioblastoma Treatment-An Update. Biomedicines.

[8] Yu Z., Gao L., Chen K., Zhang W., Zhang Q., Li Q., Hu K. (2021). Nanoparticles: A New Approach to Upgrade Cancer Diagnosis and Treatment. Nanoscale Res. Lett..

[9] Nazaruk E., Majkowska-Pilip A., Bilewicz R. (2017). Lipidic Cubic-Phase Nanoparticles-Cubosomes for Efficient Drug Delivery to Cancer Cells. Chempluschem.

[10] Yaghmur A., Mu H. (2021). Recent advances in drug delivery applications of cubosomes, hexosomes, and solid lipid nanoparticles. Acta Pharm. Sin. B.

[11] Sivadasan D., Sultan M.H., Alqahtani S.S., Javed S. (2023). Cubosomes in Drug Delivery-A Comprehensive Review on Its Structural Components, Preparation Techniques and Therapeutic Applications. Biomedicines.

[12] Johnstone R.M., Adam M., Hammond J.R., Orr L., Turbide C. (1987). Vesicle formation during reticulocyte maturation. Association of plasma membrane activities with released vesicles (exosomes). J. Biol. Chem..

[13] Moon B., Chang S. (2022). Exosome as a Delivery Vehicle for Cancer Therapy. Cells.

[14] Wu X., Wang X., Wang J., Hao Y., Liu F., Wang X., Yang L., Lu Z. (2021). The Roles of Exosomes as Future Therapeutic Agents and Diagnostic Tools for Glioma. Front. Oncol..

[15] Mukherjee A., Bisht B., Dutta S., Paul M.K. (2022). Current advances in the use of exosomes, liposomes, and bioengineered hybrid nanovesicles in cancer detection and therapy. Acta Pharmacol. Sin..

[16] Wang Z., Wang Q., Qin F., Chen J. (2024). Exosomes: A promising avenue for cancer diagnosis beyond treatment. Front. Cell Dev. Biol..

[17] Yang Z.J., Bi Q.C., Gan L.J., Zhang L.L., Wei M.J., Hong T., Liu R., Qiu C.L., Han X.J., Jiang L.P. (2022). Exosomes Derived from Glioma Cells under Hypoxia Promote Angiogenesis through Up-regulated Exosomal Connexin 43. Int. J. Med. Sci..

[18] Khatami S.H., Karami N., Taheri-Anganeh M., Taghvimi S., Tondro G., Khorsand M., Soltani Fard E., Sedighimehr N., Kazemi M., Rahimi Jaberi K. (2023). Exosomes: Promising Delivery Tools for Overcoming Blood-Brain Barrier and Glioblastoma Therapy. Mol. Neurobiol..

[19] Poinsot V., Pizzinat N., Ong-Meang V. (2024). Engineered and Mimicked Extracellular Nanovesicles for Therapeutic Delivery. Nanomaterials.

[20] Tan F., Li X., Wang Z., Li J., Shahzad K., Zheng J. (2024). Clinical applications of stem cell-derived exosomes. Signal Transduct. Target. Ther..

[21] Yadav R., Singh A.V., Kushwaha S., Chauhan D.S. (2024). Emerging role of exosomes as a liquid biopsy tool for diagnosis, prognosis & monitoring treatment response of communicable & non-communicable diseases. Indian. J. Med. Res..

[22] Balasa A., Serban G., Chinezu R., Hurghis C., Tamas F., Manu D. (2020). The Involvement of Exosomes in Glioblastoma Development, Diagnosis, Prognosis, and Treatment. Brain Sci..

[23] Osaid Z., Haider M., Hamoudi R., Harati R. (2023). Exosomes Interactions with the Blood-Brain Barrier: Implications for Cerebral Disorders and Therapeutics. Int. J. Mol. Sci..

[24] Zhang C., Song J., Lou L., Qi X., Zhao L., Fan B., Sun G., Lv Z., Fan Z., Jiao B. (2021). Doxorubicin-loaded nanoparticle coated with endothelial cells-derived exosomes for immunogenic chemotherapy of glioblastoma. Bioeng. Transl. Med..

[25] Oraiopoulou M.E., Tzamali E., Psycharakis S.E., Tzedakis G., Makatounakis T., Manolitsi K., Drakos E., Vakis A.F., Zacharakis G., Papamatheakis J. (2024). The Temozolomide-Doxorubicin paradox in Glioblastoma in vitro-in silico preclinical drug-screening. Sci. Rep..

[26] Salarpour S., Forootanfar H., Pournamdari M., Ahmadi-Zeidabadi M., Esmaeeli M., Pardakhty A. (2019). Paclitaxel incorporated exosomes derived from glioblastoma cells: Comparative study of two loading techniques. Daru.

[27] Duhamel M., Rose M., Rodet F., Murgoci A.N., Zografidou L., Regnier-Vigouroux A., Vanden Abeele F., Kobeissy F., Nataf S., Pays L. (2018). Paclitaxel Treatment and Proprotein Convertase 1/3 (PC1/3) Knockdown in Macrophages is a Promising Antiglioma Strategy as Revealed by Proteomics and Cytotoxicity Studies. Mol. Cell Proteom..

[28] Valipour E., Ranjbar F.E., Mousavi M., Ai J., Malekshahi Z.V., Mokhberian N., Taghdiri-Nooshabadi Z., Khanmohammadi M., Nooshabadi V.T. (2022). The anti-angiogenic effect of atorvastatin loaded exosomes on glioblastoma tumor cells: An in vitro 3D culture model. Microvasc. Res..

[29] Lee H., Bae K., Baek A.R., Kwon E.B., Kim Y.H., Nam S.W., Lee G.H., Chang Y. (2022). Glioblastoma-Derived Exosomes as Nanopharmaceutics for Improved Glioma Treatment. Pharmaceutics.

[30] Yin J., Zeng A., Zhang Z., Shi Z., Yan W., You Y. (2019). Exosomal transfer of miR-1238 contributes to temozolomide-resistance in glioblastoma. EBioMedicine.

[31] Kong Y.W., Ferland-McCollough D., Jackson T.J., Bushell M. (2012). microRNAs in cancer management. Lancet Oncol..

[32] Zhang Z., Guo X., Guo X., Yu R., Qian M., Wang S., Gao X., Qiu W., Guo Q., Xu J. (2021). MicroRNA-29a-3p delivery via exosomes derived from engineered human mesenchymal stem cells exerts tumour suppressive effects by inhibiting migration and vasculogenic mimicry in glioma. Aging.

[33] Kim R., Lee S., Lee J., Kim M., Kim W.J., Lee H.W., Lee M.Y., Kim J., Chang W. (2018). Exosomes derived from microRNA-584 transfected mesenchymal stem cells: Novel alternative therapeutic vehicles for cancer therapy. BMB Rep..

[34] Xu H., Zhao G., Zhang Y., Jiang H., Wang W., Zhao D., Hong J., Yu H., Qi L. (2019). Mesenchymal stem cell-derived exosomal microRNA-133b suppresses glioma progression via Wnt/beta-catenin signaling pathway by targeting EZH2. Stem Cell Res. Ther..

[35] Zhang Z., Yin J., Lu C., Wei Y., Zeng A., You Y. (2019). Exosomal transfer of long non-coding RNA SBF2-AS1 enhances chemoresistance to temozolomide in glioblastoma. J. Exp. Clin. Cancer Res..

[36] Chai Y., Wu H.T., Liang C.D., You C.Y., Xie M.X., Xiao S.W. (2020). Exosomal lncRNA ROR1-AS1 Derived from Tumor Cells Promotes Glioma Progression via Regulating miR-4686. Int. J. Nanomed..

[37] Parker Kerrigan B.C., Ledbetter D., Kronowitz M., Phillips L., Gumin J., Hossain A., Yang J., Mendt M., Singh S., Cogdell D. (2020). RNAi technology targeting the FGFR3-TACC3 fusion breakpoint: An opportunity for precision medicine. Neurooncol Adv..

[38] Haltom A.R., Hassen W.E., Hensel J., Kim J., Sugimoto H., Li B., McAndrews K.M., Conner M.R., Kirtley M.L., Luo X. (2022). Engineered exosomes targeting MYC reverse the proneural-mesenchymal transition and extend survival of glioblastoma. Extracell. Vesicle.

[39] Aqil F., Gupta R.C. (2022). Exosomes in Cancer Therapy. Cancers.

[40] Zhan Q., Yi K., Cui X., Li X., Yang S., Wang Q., Fang C., Tan Y., Li L., Xu C. (2022). Blood exosomes-based targeted delivery of cPLA2 siRNA and metformin to modulate glioblastoma energy metabolism for tailoring personalized therapy. Neuro Oncol..

[41] Rahmani R., Kiani J., Tong W.Y., Soleimani M., Voelcker N.H., Arefian E. (2023). Engineered anti-EGFRvIII targeted exosomes induce apoptosis in glioblastoma multiforme. J. Drug Target..

[42] Rehman F.U., Liu Y., Yang Q., Yang H., Liu R., Zhang D., Muhammad P., Liu Y., Hanif S., Ismail M. (2022). Heme Oxygenase-1 targeting exosomes for temozolomide resistant glioblastoma synergistic therapy. J. Control. Release.

[43] Gecys D., Kazlauskas A., Gecyte E., Pauziene N., Kulakauskiene D., Lukminaite I., Jekabsone A. (2022). Internalisation of RGD-Engineered Extracellular Vesicles by Glioblastoma Cells. Biology.

[44] Geng T., Leung E., Chamley L.W., Wu Z. (2023). Functionalisation of extracellular vesicles with cyclic-RGDyC potentially for glioblastoma targeted intracellular drug delivery. Biomater. Adv..

[45] Jia G., Han Y., An Y., Ding Y., He C., Wang X., Tang Q. (2018). NRP-1 targeted and cargo-loaded exosomes facilitate simultaneous imaging and therapy of glioma in vitro and in vivo. Biomaterials.

[46] Galardi A., De Bethlen A., Di Paolo V., Lampis S., Mastronuzzi A., Di Giannatale A. (2023). Recent Advancements on the Use of Exosomes as Drug Carriers for the Treatment of Glioblastoma. Life.

[47] Roszkowski S. (2024). Therapeutic potential of mesenchymal stem cell-derived exosomes for regenerative medicine applications. Clin. Exp. Med..

[48] Wang Y., Huo Y., Zhao C., Liu H., Shao Y., Zhu C., An L., Chen X., Chen Z. (2024). Engineered exosomes with enhanced stability and delivery efficiency for glioblastoma therapy. J. Control. Release.

[49] Akbarzadeh A., Rezaei-Sadabady R., Davaran S., Joo S.W., Zarghami N., Hanifehpour Y., Samiei M., Kouhi M., Nejati-Koshki K. (2013). Liposome: Classification, preparation, and applications. Nanoscale Res. Lett..

[50] Juhairiyah F., de Lange E.C.M. (2021). Understanding Drug Delivery to the Brain Using Liposome-Based Strategies: Studies that Provide Mechanistic Insights Are Essential. AAPS J..

[51] Picciolini S., Roda F., Gualerzi A., Mangolini V., Forleo L., Mangolini A., Sesana S., Antoniou A., Re F., Seneci P. (2023). SPRi analysis of molecular interactions of mApoE-functionalized liposomes as drug delivery systems for brain diseases. Analyst.

[52] Wang S., Yang Z., Yang C., Chen J., Zhou L., Wu Y., Lu R. (2022). Investigation of functionalised nanoplatforms using branched-ligands with different chain lengths for glioblastoma targeting. J. Drug Target..

[53] Mellinger A., Lubitz L.J., Gazaille C., Leneweit G., Bastiat G., Lepinoux-Chambaud C., Eyer J. (2023). The use of liposomes functionalized with the NFL-TBS.40-63 peptide as a targeting agent to cross the in vitro blood-brain barrier and target glioblastoma cells. Int. J. Pharm..

[54] Semyachkina-Glushkovskaya O., Shirokov A., Blokhina I., Telnova V., Vodovozova E., Alekseeva A., Boldyrev I., Fedosov I., Dubrovsky A., Khorovodov A. (2022). Intranasal Delivery of Liposomes to Glioblastoma by Photostimulation of the Lymphatic System. Pharmaceutics.

[55] Katona G., Sabir F., Sipos B., Naveed M., Schelz Z., Zupko I., Csoka I. (2022). Development of Lomustine and n-Propyl Gallate Co-Encapsulated Liposomes for Targeting Glioblastoma Multiforme via Intranasal Administration. Pharmaceutics.

[56] Song Z., Huang X., Wang J., Cai F., Zhao P., Yan F. (2021). Targeted Delivery of Liposomal Temozolomide Enhanced Anti-Glioblastoma Efficacy through Ultrasound-Mediated Blood-Brain Barrier Opening. Pharmaceutics.

[57] Mahmud H., Kasai T., Khayrani A.C., Asakura M., Oo A.K.K., Du J., Vaidyanath A., El-Ghlban S., Mizutani A., Seno A. (2018). Targeting Glioblastoma Cells Expressing CD44 with Liposomes Encapsulating Doxorubicin and Displaying Chlorotoxin-IgG Fc Fusion Protein. Int. J. Mol. Sci..

[58] Najlah M., Jain M., Wan K.W., Ahmed W., Albed Alhnan M., Phoenix D.A., Taylor K.M.G., Elhissi A. (2018). Ethanol-based proliposome delivery systems of paclitaxel for in vitro application against brain cancer cells. J. Liposome Res..

[59] Renault-Mahieux M., Vieillard V., Seguin J., Espeau P., Le D.T., Lai-Kuen R., Mignet N., Paul M., Andrieux K. (2021). Co-Encapsulation of Fisetin and Cisplatin into Liposomes for Glioma Therapy: From Formulation to Cell Evaluation. Pharmaceutics.

[60] Zhang Y., Qu H., Xue X. (2022). Blood-brain barrier penetrating liposomes with synergistic chemotherapy for glioblastoma treatment. Biomater. Sci..

[61] Wang X., Zhao Y., Dong S., Lee R.J., Yang D., Zhang H., Teng L. (2019). Cell-Penetrating Peptide and Transferrin Co-Modified Liposomes for Targeted Therapy of Glioma. Molecules.

[62] Waghule T., Laxmi Swetha K., Roy A., Narayan Saha R., Singhvi G. (2023). Exploring temozolomide encapsulated PEGylated liposomes and lyotropic liquid crystals for effective treatment of glioblastoma: In-vitro, cell line, and pharmacokinetic studies. Eur. J. Pharm. Biopharm..

[63] Ghaferi M., Raza A., Koohi M., Zahra W., Akbarzadeh A., Ebrahimi Shahmabadi H., Alavi S.E. (2022). Impact of PEGylated Liposomal Doxorubicin and Carboplatin Combination on Glioblastoma. Pharmaceutics.

[64] Shabana A.M., Xu B., Schneiderman Z., Ma J., Chen C.C., Kokkoli E. (2021). Targeted Liposomes Encapsulating miR-603 Complexes Enhance Radiation Sensitivity of Patient-Derived Glioblastoma Stem-Like Cells. Pharmaceutics.

[65] Hu Y., Jiang K., Wang D., Yao S., Lu L., Wang H., Song J., Zhou J., Fan X., Wang Y. (2022). Core-shell lipoplexes inducing active macropinocytosis promote intranasal delivery of c-Myc siRNA for treatment of glioblastoma. Acta Biomater..

[66] Grafals-Ruiz N., Rios-Vicil C.I., Lozada-Delgado E.L., Quinones-Diaz B.I., Noriega-Rivera R.A., Martinez-Zayas G., Santana-Rivera Y., Santiago-Sanchez G.S., Valiyeva F., Vivas-Mejia P.E. (2020). Brain Targeted Gold Liposomes Improve RNAi Delivery for Glioblastoma. Int. J. Nanomed..

[67] Grafals-Ruiz N., Sanchez-Alvarez A.O., Santana-Rivera Y., Lozada-Delgado E.L., Rabelo-Fernandez R.J., Rios-Vicil C.I., Valiyeva F., Vivas-Mejia P.E. (2023). MicroRNA-92b targets tumor suppressor gene FBXW7 in glioblastoma. Front. Oncol..

[68] Sun X., Chen Y., Zhao H., Qiao G., Liu M., Zhang C., Cui D., Ma L. (2018). Dual-modified cationic liposomes loaded with paclitaxel and survivin siRNA for targeted imaging and therapy of cancer stem cells in brain glioma. Drug Deliv..

[69] Wang X., Meng N., Wang S., Zhang Y., Lu L., Wang R., Ruan H., Jiang K., Wang H., Ran D. (2019). Non-immunogenic, low-toxicity and effective glioma targeting MTI-31 liposomes. J. Control. Release.

[70] Fisher C., Obaid G., Niu C., Foltz W., Goldstein A., Hasan T., Lilge L. (2019). Liposomal Lapatinib in Combination with Low-Dose Photodynamic Therapy for the Treatment of Glioma. J. Clin. Med..

[71] Moon H., Hwang K., Nam K.M., Kim Y.S., Ko M.J., Kim H.R., Lee H.J., Kim M.J., Kim T.H., Kang K.S. (2022). Enhanced delivery to brain using sonosensitive liposome and microbubble with focused ultrasound. Biomater. Adv..

[72] Semyachkina-Glushkovskaya O., Bragin D., Bragina O., Socolovski S., Shirokov A., Fedosov I., Ageev V., Blokhina I., Dubrovsky A., Telnova V. (2023). Low-Level Laser Treatment Induces the Blood-Brain Barrier Opening and the Brain Drainage System Activation: Delivery of Liposomes into Mouse Glioblastoma. Pharmaceutics.

[73] Campelo S.N., Lorenzo M.F., Partridge B., Alinezhadbalalami N., Kani Y., Garcia J., Saunier S., Thomas S.C., Hinckley J., Verbridge S.S. (2023). High-frequency irreversible electroporation improves survival and immune cell infiltration in rodents with malignant gliomas. Front. Oncol..

[74] Mainprize T., Lipsman N., Huang Y., Meng Y., Bethune A., Ironside S., Heyn C., Alkins R., Trudeau M., Sahgal A. (2019). Blood-Brain Barrier Opening in Primary Brain Tumors with Non-invasive MR-Guided Focused Ultrasound: A Clinical Safety and Feasibility Study. Sci. Rep..

[75] Papachristodoulou A., Signorell R.D., Werner B., Brambilla D., Luciani P., Cavusoglu M., Grandjean J., Silginer M., Rudin M., Martin E. (2019). Chemotherapy sensitization of glioblastoma by focused ultrasound-mediated delivery of therapeutic liposomes. J. Control. Release.

[76] Shi D., Mi G., Shen Y., Webster T.J. (2019). Glioma-targeted dual functionalized thermosensitive Ferri-liposomes for drug delivery through an in vitro blood-brain barrier. Nanoscale.

[77] Yao J., Feng X., Dai X., Peng G., Guo Z., Liu Z., Wang M., Guo W., Zhang P., Li Y. (2022). TMZ magnetic temperature-sensitive liposomes-mediated magnetothermal chemotherapy induces pyroptosis in glioblastoma. Nanomedicine.

[78] Du L., Wang P., Huang H., Li M., Roy S., Zhang Y., Guo B. (2023). Light-activatable and hyperthermia-sensitive “all-in-one” theranostics: NIR-II fluorescence imaging and chemo-photothermal therapy of subcutaneous glioblastoma by temperature-sensitive liposome-containing AIEgens and paclitaxel. Front. Bioeng. Biotechnol..

[79] Luderer M.J., Muz B., Alhallak K., Sun J., Wasden K., Guenthner N., de la Puente P., Federico C., Azab A.K. (2019). Thermal Sensitive Liposomes Improve Delivery of Boronated Agents for Boron Neutron Capture Therapy. Pharm. Res..

[80] Kanygin V., Zaboronok A., Taskaeva I., Zavjalov E., Mukhamadiyarov R., Kichigin A., Kasatova A., Razumov I., Sibirtsev R., Mathis B.J. (2021). In Vitro and In Vivo Evaluation of Fluorescently Labeled Borocaptate-Containing Liposomes. J. Fluoresc..

[81] Zavjalov E., Zaboronok A., Kanygin V., Kasatova A., Kichigin A., Mukhamadiyarov R., Razumov I., Sycheva T., Mathis B.J., Maezono S.E.B. (2020). Accelerator-based boron neutron capture therapy for malignant glioma: A pilot neutron irradiation study using boron phenylalanine, sodium borocaptate and liposomal borocaptate with a heterotopic U87 glioblastoma model in SCID mice. Int. J. Radiat. Biol..

[82] Xu H., Li C., Wei Y., Zheng H., Zheng H., Wang B., Piao J.G., Li F. (2021). Angiopep-2-modified calcium arsenite-loaded liposomes for targeted and pH-responsive delivery for anti-glioma therapy. Biochem. Biophys. Res. Commun..

[83] Kim J.S., Shin D.H., Kim J.S. (2018). Dual-targeting immunoliposomes using angiopep-2 and CD133 antibody for glioblastoma stem cells. J. Control. Release.

[84] Ismail M., Yang W., Li Y., Chai T., Zhang D., Du Q., Muhammad P., Hanif S., Zheng M., Shi B. (2022). Targeted liposomes for combined delivery of artesunate and temozolomide to resistant glioblastoma. Biomaterials.

[85] Pizzocri M., Re F., Stanzani E., Formicola B., Tamborini M., Lauranzano E., Ungaro F., Rodighiero S., Francolini M., Gregori M. (2021). Radiation and adjuvant drug-loaded liposomes target glioblastoma stem cells and trigger in-situ immune response. Neurooncol. Adv..

[86] Ouyang J., Jiang Y., Deng C., Zhong Z., Lan Q. (2021). Doxorubicin Delivered via ApoE-Directed Reduction-Sensitive Polymersomes Potently Inhibit Orthotopic Human Glioblastoma Xenografts in Nude Mice. Int. J. Nanomed..

[87] Aguiar S.I., Dias J.N.R., Andre A.S., Silva M.L., Martins D., Carrapico B., Castanho M., Carrico J., Cavaco M., Gaspar M.M. (2021). Highly Specific Blood-Brain Barrier Transmigrating Single-Domain Antibodies Selected by an In Vivo Phage Display Screening. Pharmaceutics.

[88] Aguilar-Perez K.M., Aviles-Castrillo J.I., Medina D.I., Parra-Saldivar R., Iqbal H.M.N. (2020). Insight Into Nanoliposomes as Smart Nanocarriers for Greening the Twenty-First Century Biomedical Settings. Front. Bioeng. Biotechnol..

[89] Wang Y., Lv S., Cao F., Ding Z., Liu J., Chen Q., Gao J., Huang X. (2022). Investigations on the influence of the structural flexibility of nanoliposomes on their properties. J. Liposome Res..

[90] Wang Y., Ding Z., Lv S., Liu J., Pan J., Yu Y., Gao J., Huang X. (2023). Development of tLyP-1 functionalized nanoliposomes with tunable internal water phase for glioma targeting. J. Liposome Res..

[91] Zhao M., Zhao M., Fu C., Yu Y., Fu A. (2018). Targeted therapy of intracranial glioma model mice with curcumin nanoliposomes. Int. J. Nanomed..

[92] Mendanha D., Gimondi S., Costa B.M., Ferreira H., Neves N.M. (2023). Microfluidic-derived docosahexaenoic acid liposomes for glioblastoma therapy. Nanomedicine.

[93] Luiz M.T., Viegas J.S.R., Abriata J.P., Tofani L.B., Vaidergorn M.M., Emery F.D.S., Chorilli M., Marchetti J.M. (2021). Docetaxel-loaded folate-modified TPGS-transfersomes for glioblastoma multiforme treatment. Mater. Sci. Eng. C Mater. Biol. Appl..

[94] Tedesco A.C., Silva E.P.O., Jayme C.C., Piva H.L., Franchi L.P. (2021). Cholesterol-rich nanoemulsion (LDE) as a novel drug delivery system to diagnose, delineate, and treat human glioblastoma. Mater. Sci. Eng. C Mater. Biol. Appl..

[95] Pavlov R., Romanova E., Kuznetsov D., Lyubina A., Amerhanova S., Voloshina A., Buzyurova D., Babaev V., Zueva I., Petrov K. (2023). The Formation of Morphologically Stable Lipid Nanocarriers for Glioma Therapy. Int. J. Mol. Sci..

[96] Jia Y., Sheng Z., Hu D., Yan F., Zhu M., Gao G., Wang P., Liu X., Wang X., Zheng H. (2018). Highly penetrative liposome nanomedicine generated by a biomimetic strategy for enhanced cancer chemotherapy. Biomater. Sci..

[97] Perini G., Giulimondi F., Palmieri V., Augello A., Digiacomo L., Quagliarini E., Pozzi D., Papi M., Caracciolo G. (2021). Inhibiting the Growth of 3D Brain Cancer Models with Bio-Coronated Liposomal Temozolomide. Pharmaceutics.

[98] Elinzano H., Toms S., Robison J., Mohler A., Carcieri A., Cielo D., Donnelly J., Disano D., Vatketich J., Baekey J. (2021). Nanoliposomal Irinotecan and Metronomic Temozolomide for Patients With Recurrent Glioblastoma: BrUOG329, A Phase I Brown University Oncology Research Group Trial. Am. J. Clin. Oncol..

[99] Kasenda B., Konig D., Manni M., Ritschard R., Duthaler U., Bartoszek E., Barenwaldt A., Deuster S., Hutter G., Cordier D. (2022). Targeting immunoliposomes to EGFR-positive glioblastoma. ESMO Open.

[100] Beier C.P., Schmid C., Gorlia T., Kleinletzenberger C., Beier D., Grauer O., Steinbrecher A., Hirschmann B., Brawanski A., Dietmaier C. (2009). RNOP-09: Pegylated liposomal doxorubicine and prolonged temozolomide in addition to radiotherapy in newly diagnosed glioblastoma--a phase II study. BMC Cancer.

[101] Whittle J.R., Lickliter J.D., Gan H.K., Scott A.M., Simes J., Solomon B.J., MacDiarmid J.A., Brahmbhatt H., Rosenthal M.A. (2015). First in human nanotechnology doxorubicin delivery system to target epidermal growth factor receptors in recurrent glioblastoma. J. Clin. Neurosci..

[102] Chastagner P., Devictor B., Geoerger B., Aerts I., Leblond P., Frappaz D., Gentet J.C., Bracard S., André N. (2015). Phase I study of non-pegylated liposomal doxorubicin in children with recurrent/refractory high-grade glioma. Cancer Chemother. Pharmacol..

[103] Nazaruk E., Majkowska-Pilip A., Godlewska M., Salamończyk M., Gawel D. (2018). Electrochemical and biological characterization of lyotropic liquid crystalline phases—Retardation of drug release from hexagonal mesophases. J. Electroanal. Chem..

[104] Zhang L., Li J., Tian D., Sun L., Wang X., Tian M. (2020). Theranostic combinatorial drug-loaded coated cubosomes for enhanced targeting and efficacy against cancer cells. Cell Death Dis..

[105] Clogston J., Caffrey M. (2005). Controlling release from the lipidic cubic phase. Amino acids, peptides, proteins and nucleic acids. J. Control. Release.

[106] Dully M., Brasnett C., Djeghader A., Seddon A., Neilan J., Murray D., Butler J., Soulimane T., Hudson S.P. (2020). Modulating the release of pharmaceuticals from lipid cubic phases using a lipase inhibitor. J. Colloid. Interface Sci..

[107] Azhari H., Younus M., Hook S.M., Boyd B.J., Rizwan S.B. (2021). Cubosomes enhance drug permeability across the blood-brain barrier in zebrafish. Int. J. Pharm..

[108] Gajda E., Godlewska M., Mariak Z., Nazaruk E., Gawel D. (2020). Combinatory Treatment with miR-7-5p and Drug-Loaded Cubosomes Effectively Impairs Cancer Cells. Int. J. Mol. Sci..

[109] Flak D.K., Adamski V., Nowaczyk G., Szutkowski K., Synowitz M., Jurga S., Held-Feindt J. (2020). AT101-Loaded Cubosomes as an Alternative for Improved Glioblastoma Therapy. Int. J. Nanomed..

[110] Cai X., Refaat A., Gan P.Y., Fan B., Yu H., Thang S.H., Drummond C.J., Voelcker N.H., Tran N., Zhai J. (2024). Angiopep-2-Functionalized Lipid Cubosomes for Blood-Brain Barrier Crossing and Glioblastoma Treatment. ACS Appl. Mater. Interfaces.

[111] Zielinska A., Carreiro F., Oliveira A.M., Neves A., Pires B., Venkatesh D.N., Durazzo A., Lucarini M., Eder P., Silva A.M. (2020). Polymeric Nanoparticles: Production, Characterization, Toxicology and Ecotoxicology. Molecules.

[112] Tang C., Wang Y., Wu M., Wang Z., Zhou Y., Lin Y., Wang Y., Xu H. (2024). Cancer cell membrane-camouflaged biomimetic nanoparticles for enhancing chemo-radiation therapy efficacy in glioma. J. Biomed. Res..

[113] Lopez-Bertoni H., Kozielski K.L., Rui Y., Lal B., Vaughan H., Wilson D.R., Mihelson N., Eberhart C.G., Laterra J., Green J.J. (2018). Bioreducible Polymeric Nanoparticles Containing Multiplexed Cancer Stem Cell Regulating miRNAs Inhibit Glioblastoma Growth and Prolong Survival. Nano Lett..

[114] Silverman L., Bhatti G., Wulff J.E., Moffitt M.G. (2022). Improvements in Drug-Delivery Properties by Co-Encapsulating Curcumin in SN-38-Loaded Anticancer Polymeric Nanoparticles. Mol. Pharm..

[115] Guerrero-Cazares H., Tzeng S.Y., Young N.P., Abutaleb A.O., Quinones-Hinojosa A., Green J.J. (2014). Biodegradable polymeric nanoparticles show high efficacy and specificity at DNA delivery to human glioblastoma in vitro and in vivo. ACS Nano.

[116] Ferreira N.N., Granja S., Boni F.I., Ferreira L.M.B., Reis R.M., Baltazar F., Gremiao M.P.D. (2020). A novel strategy for glioblastoma treatment combining alpha-cyano-4-hydroxycinnamic acid with cetuximab using nanotechnology-based delivery systems. Drug Deliv. Transl. Res..

[117] Shetty K., Yadav K.S. (2024). Temozolomide nano-in-nanofiber delivery system with sustained release and enhanced cellular uptake by U87MG cells. Drug Dev. Ind. Pharm..

[118] Solano A.G., Dupuy J., Therriault H., Liberelle B., Faucheux N., Lauzon M.A., Virgilio N., Paquette B. (2021). An alginate-based macroporous hydrogel matrix to trap cancer cells. Carbohydr. Polym..

[119] Lu Y., Cheng D., Niu B., Wang X., Wu X., Wang A. (2023). Properties of Poly (Lactic-co-Glycolic Acid) and Progress of Poly (Lactic-co-Glycolic Acid)-Based Biodegradable Materials in Biomedical Research. Pharmaceuticals.

[120] Ye Z., Gao L., Cai J., Wang Y., Li Y., Tong S., Yan T., Sun Q., Qi Y., Xu Y. (2022). Esterase-responsive and size-optimized prodrug nanoparticles for effective intracranial drug delivery and glioblastoma treatment. Nanomedicine.

[121] Ferreira N.N., Granja S., Boni F.I., Prezotti F.G., Ferreira L.M.B., Cury B.S.F., Reis R.M., Baltazar F., Gremiao M.P.D. (2020). Modulating chitosan-PLGA nanoparticle properties to design a co-delivery platform for glioblastoma therapy intended for nose-to-brain route. Drug Deliv. Transl. Res..

[122] Malekpour M.R., Hosseindoost S., Madani F., Kamali M., Khosravani M., Adabi M. (2023). Combination nanochemotherapy of brain tumor using polymeric nanoparticles loaded with doxorubicin and paclitaxel: An in vitro and in vivo study. Eur. J. Pharm. Biopharm..

[123] Schmitt R.R., Mahajan S.D., Pliss A., Prasad P.N. (2022). Small molecule based EGFR targeting of biodegradable nanoparticles containing temozolomide and Cy5 dye for greatly enhanced image-guided glioblastoma therapy. Nanomedicine.

[124] Caban-Toktas S., Sahin A., Lule S., Esendagli G., Vural I., Karli Oguz K., Soylemezoglu F., Mut M., Dalkara T., Khan M. (2020). Combination of Paclitaxel and R-flurbiprofen loaded PLGA nanoparticles suppresses glioblastoma growth on systemic administration. Int. J. Pharm..

[125] Younis M., Shaikh S., Shahzad K.A., Tan F., Wang Z., Lashari M.H. (2024). Amrubicin encapsulated PLGA NPs inhibits the PI3K/AKT signaling pathway by activating PTEN and inducing apoptosis in TMZ-resistant Glioma. Biomed. Mater..

[126] Maliyakkal N., Appadath Beeran A., Udupa N. (2021). Nanoparticles of cisplatin augment drug accumulations and inhibit multidrug resistance transporters in human glioblastoma cells. Saudi Pharm. J..

[127] Kaur J., Jakhmola S., Singh R.R., Joshi B., Jha H.C., Joshi A. (2021). Ultrasonic Atomizer-Driven Development of Biocompatible and Biodegradable Poly(d,l-lactide-co-glycolide) Nanocarrier-Encapsulated Suberoylanilide Hydroxamic Acid to Combat Brain Cancer. ACS Appl. Bio Mater..

[128] Kuzminska J., Sobczak A., Majchrzak-Celinska A., Zolnowska I., Gostynska A., Jadach B., Krajka-Kuzniak V., Jelinska A., Stawny M. (2023). Etoricoxib-Cannabidiol Combo: Potential Role in Glioblastoma Treatment and Development of PLGA-Based Nanoparticles. Pharmaceutics.

[129] Kannappan V., Liu Y., Wang Z., Azar K., Kurusamy S., Kilari R.S., Armesilla A.L., Morris M.R., Najlah M., Liu P. (2022). PLGA-Nano-Encapsulated Disulfiram Inhibits Hypoxia-Induced NF-kappaB, Cancer Stem Cells, and Targets Glioblastoma In Vitro and In Vivo. Mol. Cancer Ther..

[130] Ramalho M.J., Bravo M., Loureiro J.A., Lima J., Pereira M.C. (2022). Transferrin-modified nanoparticles for targeted delivery of Asiatic acid to glioblastoma cells. Life Sci..

[131] Ramalho M.J., Torres I.D., Loureiro J.A., Lima J., Pereira M.C. (2023). Transferrin-Conjugated PLGA Nanoparticles for Co-Delivery of Temozolomide and Bortezomib to Glioblastoma Cells. ACS Appl. Nano Mater..

[132] Ghoreyshi N., Ghahremanloo A., Javid H., Homayouni Tabrizi M., Hashemy S.I. (2023). Effect of folic acid-linked chitosan-coated PLGA-based curcumin nanoparticles on the redox system of glioblastoma cancer cells. Phytochem. Anal..

[133] Vasey C.E., Cavanagh R.J., Taresco V., Moloney C., Smith S., Rahman R., Alexander C. (2021). Polymer Pro-Drug Nanoparticles for Sustained Release of Cytotoxic Drugs Evaluated in Patient-Derived Glioblastoma Cell Lines and In Situ Gelling Formulations. Pharmaceutics.

[134] Iqbal S., Martins A.F., Sohail M., Zhao J., Deng Q., Li M., Zhao Z. (2022). Synthesis and Characterization of Poly (beta-amino Ester) and Applied PEGylated and Non-PEGylated Poly (beta-amino ester)/Plasmid DNA Nanoparticles for Efficient Gene Delivery. Front. Pharmacol..

[135] Tzeng S.Y., Wilson D.R., Hansen S.K., Quinones-Hinojosa A., Green J.J. (2016). Polymeric nanoparticle-based delivery of TRAIL DNA for cancer-specific killing. Bioeng. Transl. Med..

[136] Kim J., Mondal S.K., Tzeng S.Y., Rui Y., Al-Kharboosh R., Kozielski K.K., Bhargav A.G., Garcia C.A., Quinones-Hinojosa A., Green J.J. (2020). Poly(ethylene glycol)-Poly(beta-amino ester)-Based Nanoparticles for Suicide Gene Therapy Enhance Brain Penetration and Extend Survival in a Preclinical Human Glioblastoma Orthotopic Xenograft Model. ACS Biomater. Sci. Eng..

[137] Kozielski K.L., Ruiz-Valls A., Tzeng S.Y., Guerrero-Cazares H., Rui Y., Li Y., Vaughan H.J., Gionet-Gonzales M., Vantucci C., Kim J. (2019). Cancer-selective nanoparticles for combinatorial siRNA delivery to primary human GBM in vitro and in vivo. Biomaterials.

[138] Coudane J., Nottelet B., Mouton J., Garric X., Van Den Berghe H. (2022). Poly(epsilon-caprolactone)-Based Graft Copolymers: Synthesis Methods and Applications in the Biomedical Field: A Review. Molecules.

[139] Gherardini L., Vetri Buratti V., Maturi M., Inzalaco G., Locatelli E., Sambri L., Gargiulo S., Barone V., Bonente D., Bertelli E. (2023). Loco-regional treatment with temozolomide-loaded thermogels prevents glioblastoma recurrences in orthotopic human xenograft models. Sci. Rep..

[140] Aranaz I., Alcantara A.R., Civera M.C., Arias C., Elorza B., Heras Caballero A., Acosta N. (2021). Chitosan: An Overview of Its Properties and Applications. Polymers.

[141] Sabourian P., Ji J., Lotocki V., Moquin A., Hanna R., Frounchi M., Maysinger D., Kakkar A. (2020). Facile design of autogenous stimuli-responsive chitosan/hyaluronic acid nanoparticles for efficient small molecules to protein delivery. J. Mater. Chem. B.

[142] Sathiyaseelan A., Saravanakumar K., Jayalakshmi J., Gopi M., Shajahan A., Barathikannan K., Kalaichelvan P.T., Wang M.H. (2020). Trigonelline-loaded chitosan nanoparticles prompted antitumor activity on glioma cells and biocompatibility with pheochromocytoma cells. Int. J. Biol. Macromol..

[143] Alswailem R., Alqahtani F.Y., Aleanizy F.S., Alrfaei B.M., Badran M., Alqahtani Q.H., Abdelhady H.G., Alsarra I. (2022). MicroRNA-219 loaded chitosan nanoparticles for treatment of glioblastoma. Artif. Cells Nanomed. Biotechnol..

[144] Fukui N., Yawata T., Nakajo T., Kawanishi Y., Higashi Y., Yamashita T., Aratake T., Honke K., Ueba T. (2020). Targeting CD146 using folic acid-conjugated nanoparticles and suppression of tumor growth in a mouse glioma model. J. Neurosurg..

[145] Song P., Song N., Li L., Wu M., Lu Z., Zhao X. (2021). Angiopep-2-Modified Carboxymethyl Chitosan-Based pH/Reduction Dual-Stimuli-Responsive Nanogels for Enhanced Targeting Glioblastoma. Biomacromolecules.

[146] Trivedi S., Belgamwar V. (2024). Fabrication and optimization of chitosan-g-m-PEG-NH(2) copolymer for advanced glioblastoma therapy using surface engineered lentinan loaded nanovesicles for nasal delivery. Int. J. Biol. Macromol..

[147] Gabold B., Adams F., Brameyer S., Jung K., Ried C.L., Merdan T., Merkel O.M. (2023). Transferrin-modified chitosan nanoparticles for targeted nose-to-brain delivery of proteins. Drug Deliv. Transl. Res..

[148] Van Bavel N., Lewrenz A.M., Issler T., Pang L., Anikovskiy M., Prenner E.J. (2023). Synthesis of Alginate Nanoparticles Using Hydrolyzed and Enzyme-Digested Alginate Using the Ionic Gelation and Water-in-Oil Emulsion Method. Polymers.

[149] Madkhali O.A. (2023). Drug Delivery of Gelatin Nanoparticles as a Biodegradable Polymer for the Treatment of Infectious Diseases: Perspectives and Challenges. Polymers.

[150] Chen C., Fan R., Wang Y., Wang L., Huang C., Zhou L., Xu J., Chen H., Guo G. (2021). Hyaluronic Acid-Conjugated Nanoparticles for the Targeted Delivery of Cabazitaxel to CD44-Overexpressing Glioblastoma Cells. J. Biomed. Nanotechnol..

[151] Lubanska D., Alrashed S., Mason G.T., Nadeem F., Awada A., DiPasquale M., Sorge A., Malik A., Kojic M., Soliman M.A.R. (2022). Impairing proliferation of glioblastoma multiforme with CD44+ selective conjugated polymer nanoparticles. Sci. Rep..

[152] Ibarra L.E., Beauge L., Arias-Ramos N., Rivarola V.A., Chesta C.A., Lopez-Larrubia P., Palacios R.E. (2020). Trojan horse monocyte-mediated delivery of conjugated polymer nanoparticles for improved photodynamic therapy of glioblastoma. Nanomed..

[153] Elgiddawy N., Elnagar N., Korri-Youssoufi H., Yassar A. (2023). pi-Conjugated Polymer Nanoparticles from Design, Synthesis to Biomedical Applications: Sensing, Imaging, and Therapy. Microorganisms.

[154] Pecher J., Mecking S. (2010). Nanoparticles of Conjugated Polymers. Chem. Rev..

[155] Caverzán M.D., Beaugé L., Chesta C.A., Palacios R.E., Ibarra L.E. (2020). Photodynamic therapy of Glioblastoma cells using doped conjugated polymer nanoparticles: An in vitro comparative study based on redox status. J. Photochem. Photobiol. B Biol..

[156] Liang Y., Li Z., Yuan H., Wang L., Gao L.H. (2021). Poly(p-phenylenevinylene) nanoparticles modified with antiEGFRvIII for specific glioblastoma therapy. Sci. Rep..

[157] Caverzan M.D., Oliveda P.M., Beauge L., Palacios R.E., Chesta C.A., Ibarra L.E. (2023). Metronomic Photodynamic Therapy with Conjugated Polymer Nanoparticles in Glioblastoma Tumor Microenvironment. Cells.

[158] Han L., Kong D.K., Zheng M.Q., Murikinati S., Ma C., Yuan P., Li L., Tian D., Cai Q., Ye C. (2016). Increased Nanoparticle Delivery to Brain Tumors by Autocatalytic Priming for Improved Treatment and Imaging. ACS Nano.

[159] Wu H., Gao X., Luo Y., Yu J., Long G., Jiang Z., Zhou J. (2022). Targeted Delivery of Chemo-Sonodynamic Therapy via Brain Targeting, Glutathione-Consumable Polymeric Nanoparticles for Effective Brain Cancer Treatment. Adv. Sci..

[160] Wang Q., Li J.M., Yu H., Deng K., Zhou W., Wang C.X., Zhang Y., Li K.H., Zhuo R.X., Huang S.W. (2018). Fluorinated polymeric micelles to overcome hypoxia and enhance photodynamic cancer therapy. Biomater. Sci..

[161] Xu H.L., Fan Z.L., ZhuGe D.L., Tong M.Q., Shen B.X., Lin M.T., Zhu Q.Y., Jin B.H., Sohawon Y., Yao Q. (2018). Ratiometric delivery of two therapeutic candidates with inherently dissimilar physicochemical property through pH-sensitive core-shell nanoparticles targeting the heterogeneous tumor cells of glioma. Drug Deliv..

[162] Goppert N.E., Quader S., Van Guyse J.F.R., Weber C., Kataoka K., Schubert U.S. (2023). Amphiphilic Poly(2-oxazoline)s with Glycine-Containing Hydrophobic Blocks Tailored for Panobinostat- and Imatinib-Loaded Micelles. Biomacromolecules.

[163] Wang M., Malfanti A., Bastiancich C., Preat V. (2023). Synergistic effect of doxorubicin lauroyl hydrazone derivative delivered by alpha-tocopherol succinate micelles for the treatment of glioblastoma. Int. J. Pharm. X.

[164] Chauhan P.S., Kumarasamy M., Carcaboso A.M., Sosnik A., Danino D. (2021). Multifunctional silica-coated mixed polymeric micelles for integrin-targeted therapy of pediatric patient-derived glioblastoma. Mater. Sci. Eng. C Mater. Biol. Appl..

[165] Xu W., Ling P., Zhang T. (2013). Polymeric micelles, a promising drug delivery system to enhance bioavailability of poorly water-soluble drugs. J. Drug Deliv..

[166] Kanazawa T., Taki H., Okada H. (2020). Nose-to-brain drug delivery system with ligand/cell-penetrating peptide-modified polymeric nano-micelles for intracerebral gliomas. Eur. J. Pharm. Biopharm..

[167] Wu C., Xu Q., Chen X., Liu J. (2019). Delivery luteolin with folacin-modified nanoparticle for glioma therapy. Int. J. Nanomed..

[168] Lu L., Zhao X., Fu T., Li K., He Y., Luo Z., Dai L., Zeng R., Cai K. (2020). An iRGD-conjugated prodrug micelle with blood-brain-barrier penetrability for anti-glioma therapy. Biomaterials.

[169] Suzuki K., Miura Y., Mochida Y., Miyazaki T., Toh K., Anraku Y., Melo V., Liu X., Ishii T., Nagano O. (2019). Glucose transporter 1-mediated vascular translocation of nanomedicines enhances accumulation and efficacy in solid tumors. J. Control. Release.

[170] Guo X., Wu G., Wang H., Chen L. (2019). Pep-1&borneol-Bifunctionalized Carmustine-Loaded Micelles Enhance Anti-Glioma Efficacy Through Tumor-Targeting and BBB-Penetrating. J. Pharm. Sci..

[171] Smiley S.B., Yun Y., Ayyagari P., Shannon H.E., Pollok K.E., Vannier M.W., Das S.K., Veronesi M.C. (2021). Development of CD133 Targeting Multi-Drug Polymer Micellar Nanoparticles for Glioblastoma—In Vitro Evaluation in Glioblastoma Stem Cells. Pharm. Res..

[172] Greish K., Jasim A., Parayath N., Abdelghany S., Alkhateeb A., Taurin S., Nehoff H. (2018). Micellar formulations of Crizotinib and Dasatinib in the management of glioblastoma multiforme. J. Drug Target..

[173] De A., Kang J.H., Sauraj, Lee O.H., Ko Y.T. (2024). Optimizing long-term stability of siRNA using thermoassemble ionizable reverse pluronic-Bcl2 micelleplexes. Int. J. Biol. Macromol..

[174] Tan X., Kim G., Lee D., Oh J., Kim M., Piao C., Lee J., Lee M.S., Jeong J.H., Lee M. (2018). A curcumin-loaded polymeric micelle as a carrier of a microRNA-21 antisense-oligonucleotide for enhanced anti-tumor effects in a glioblastoma animal model. Biomater. Sci..

[175] Tian Y., Mi G., Chen Q., Chaurasiya B., Li Y., Shi D., Zhang Y., Webster T.J., Sun C., Shen Y. (2020). Correction to Acid-Induced Activated Cell-Penetrating Peptide-Modified Cholesterol-Conjugated Polyoxyethylene Sorbitol Oleate Mixed Micelles for pH-Triggered Drug Release and Efficient Brain Tumor Targeting Based on Charge Reversal Mechanism. ACS Appl. Mater. Interfaces.

[176] Wang P., Yu N., Wang Y., Sun H., Yang Z., Zhou S. (2018). Co-delivery of PLK1-specific shRNA and doxorubicin via core-crosslinked pH-sensitive and redox ultra-sensitive micelles for glioma therapy. J. Mater. Chem. B.

[177] Peng Y., Huang J., Xiao H., Wu T., Shuai X. (2018). Codelivery of temozolomide and siRNA with polymeric nanocarrier for effective glioma treatment. Int. J. Nanomed..

[178] Shi H., Sun S., Xu H., Zhao Z., Han Z., Jia J., Wu D., Lu J., Liu H., Yu R. (2020). Combined Delivery of Temozolomide and siPLK1 Using Targeted Nanoparticles to Enhance Temozolomide Sensitivity in Glioma. Int. J. Nanomed..

[179] Liu Y., Dai S., Wen L., Zhu Y., Tan Y., Qiu G., Meng T., Yu F., Yuan H., Hu F. (2020). Enhancing Drug Delivery for Overcoming Angiogenesis and Improving the Phototherapy Efficacy of Glioblastoma by ICG-Loaded Glycolipid-Like Micelles. Int. J. Nanomed..

[180] Liu H., Wang X., Huang Y., Li H., Peng C., Yang H., Li J., Hong H., Lei Z., Zhang X. (2019). Biocompatible Croconaine Aggregates with Strong 1.2-1.3 mum Absorption for NIR-IIa Photoacoustic Imaging in Vivo. ACS Appl. Mater. Interfaces.

[181] Jiao X., Yu Y., Meng J., He M., Zhang C.J., Geng W., Ding B., Wang Z., Ding X. (2019). Dual-targeting and microenvironment-responsive micelles as a gene delivery system to improve the sensitivity of glioma to radiotherapy. Acta Pharm. Sin. B.

[182] Zhang S., Jiao X., Heger M., Gao S., He M., Xu N., Zhang J., Zhang M., Yu Y., Ding B. (2022). A tumor microenvironment-responsive micelle co-delivered radiosensitizer Dbait and doxorubicin for the collaborative chemo-radiotherapy of glioblastoma. Drug Deliv..

[183] Kinoh H., Quader S., Shibasaki H., Liu X., Maity A., Yamasoba T., Cabral H., Kataoka K. (2020). Translational Nanomedicine Boosts Anti-PD1 Therapy to Eradicate Orthotopic PTEN-Negative Glioblastoma. ACS Nano.

[184] Zhang Z., Xu X., Du J., Chen X., Xue Y., Zhang J., Yang X., Chen X., Xie J., Ju S. (2024). Redox-responsive polymer micelles co-encapsulating immune checkpoint inhibitors and chemotherapeutic agents for glioblastoma therapy. Nat. Commun..

[185] Li J., Du Y., Jiang Z., Tian Y., Qiu N., Wang Y., Lqbal M.Z., Hu M., Zou R., Luo L. (2018). Y(1) receptor ligand-based nanomicelle as a novel nanoprobe for glioma-targeted imaging and therapy. Nanoscale.

[186] Groult H., Garcia-Alvarez I., Romero-Ramirez L., Nieto-Sampedro M., Herranz F., Fernandez-Mayoralas A., Ruiz-Cabello J. (2018). Micellar Iron Oxide Nanoparticles Coated with Anti-Tumor Glycosides. Nanomaterials.

[187] Tan J., Duan X., Zhang F., Ban X., Mao J., Cao M., Han S., Shuai X., Shen J. (2020). Theranostic Nanomedicine for Synergistic Chemodynamic Therapy and Chemotherapy of Orthotopic Glioma. Adv. Sci..

[188] Wang X., Liu G., Chen N., Wu J., Zhang J., Qian Y., Zhang L., Zhou D., Yu Y. (2020). Angiopep2-Conjugated Star-Shaped Polyprodrug Amphiphiles for Simultaneous Glioma-Targeting Therapy and MR Imaging. ACS Appl. Mater. Interfaces.

[189] Li X., Shi L., Li Y., Li Q., Duan X., Wang Y., Li Q. (2020). The enhanced treatment efficacy of invasive brain glioma by dual-targeted artemether plus paclitaxel micelles. Artif. Cells Nanomed. Biotechnol..

[190] Wang K., Zhao B., Ao Y., Zhu J., Zhao C., Wang W., Zou Y., Huang D., Zhong Y., Chen W. (2023). Super-small zwitterionic micelles enable the improvement of blood-brain barrier crossing for efficient orthotopic glioblastoma combinational therapy. J. Control. Release.

[191] Gleason J.M., Klass S.H., Huang P., Ozawa T., Santos R.A., Fogarty M.M., Raleigh D.R., Berger M.S., Francis M.B. (2022). Intrinsically Disordered Protein Micelles as Vehicles for Convection-Enhanced Drug Delivery to Glioblastoma Multiforme. ACS Appl. Bio Mater..

[192] Mao K., Jiang Q., Jiang Y., Fu Z., Hu J., Sun H., Mao W. (2023). Ultra-small micelles together with UTMD enhanced the therapeutic effect of docetaxel on Glioblastoma. Eur. J. Pharm. Sci..

[193] Wei J., Xia Y., Meng F., Ni D., Qiu X., Zhong Z. (2021). Small, Smart, and LDLR-Specific Micelles Augment Sorafenib Therapy of Glioblastoma. Biomacromolecules.

[194] Street S.T.G., Chrenek J., Harniman R.L., Letwin K., Mantell J.M., Borucu U., Willerth S.M., Manners I. (2022). Length-Controlled Nanofiber Micelleplexes as Efficient Nucleic Acid Delivery Vehicles. J. Am. Chem. Soc..

[195] Lotocki V., Yazdani H., Zhang Q., Gran E.R., Nyrko A., Maysinger D., Kakkar A. (2021). Miktoarm Star Polymers with Environment-Selective ROS/GSH Responsive Locations: From Modular Synthesis to Tuned Drug Release through Micellar Partial Corona Shedding and/or Core Disassembly. Macromol. Biosci..

[196] Torcasio S.M., Oliva R., Montesi M., Panseri S., Bassi G., Mazzaglia A., Piperno A., Coulembier O., Scala A. (2022). Three-armed RGD-decorated starPLA-PEG nanoshuttle for docetaxel delivery. Biomater. Adv..

[197] Jung B.T., Jung K., Lim M., Li M., Santos R., Ozawa T., Xu T. (2021). Design of 18 nm Doxorubicin-Loaded 3-Helix Micelles: Cellular Uptake and Cytotoxicity in Patient-Derived GBM6 Cells. ACS Biomater. Sci. Eng..

[198] Jiang T., Qiao Y., Ruan W., Zhang D., Yang Q., Wang G., Chen Q., Zhu F., Yin J., Zou Y. (2021). Cation-Free siRNA Micelles as Effective Drug Delivery Platform and Potent RNAi Nanomedicines for Glioblastoma Therapy. Adv. Mater..

[199] Porret E., Hoang S., Denis C., Doris E., Hruby M., Novell A., Gravel E., Truillet C. (2023). Sonoporation-assisted micelle delivery in subcutaneous glioma-bearing mice evaluated by PET/fluorescent bi-modal imaging. Nanoscale.

[200] Maciejewski M. (1982). Concepts of trapping topologically by shell molecules. J. Macromol. Sci.—Chem..

[201] Liaw K., Zhang F., Mangraviti A., Kannan S., Tyler B., Kannan R.M. (2020). Dendrimer size effects on the selective brain tumor targeting in orthotopic tumor models upon systemic administration. Bioeng. Transl. Med..

[202] Abbasi E., Aval S.F., Akbarzadeh A., Milani M., Nasrabadi H.T., Joo S.W., Hanifehpour Y., Nejati-Koshki K., Pashaei-Asl R. (2014). Dendrimers: Synthesis, applications, and properties. Nanoscale Res. Lett..

[203] Garrigue P., Tang J., Ding L., Bouhlel A., Tintaru A., Laurini E., Huang Y., Lyu Z., Zhang M., Fernandez S. (2018). Self-assembling supramolecular dendrimer nanosystem for PET imaging of tumors. Proc. Natl. Acad. Sci. USA.

[204] Sharma A., Sharma R., Zhang Z., Liaw K., Kambhampati S.P., Porterfield J.E., Lin K.C., DeRidder L.B., Kannan S., Kannan R.M. (2020). Dense hydroxyl polyethylene glycol dendrimer targets activated glia in multiple CNS disorders. Sci. Adv..

[205] Baldwin E.L., Osheroff N. (2005). Etoposide, topoisomerase II and cancer. Curr. Med. Chem. Anticancer. Agents.

[206] Wrobel K., Wolowiec S., Markowicz J., Walajtys-Rode E., Uram L. (2022). Synthesis of Biotinylated PAMAM G3 Dendrimers Substituted with R-Glycidol and Celecoxib/Simvastatin as Repurposed Drugs and Evaluation of Their Increased Additive Cytotoxicity for Cancer Cell Lines. Cancers.

[207] Lin M.H., Chang L.C., Chung C.Y., Huang W.C., Lee M.H., Chen K.T., Lai P.S., Yang J.T. (2021). Photochemical Internalization of Etoposide Using Dendrimer Nanospheres Loaded with Etoposide and Protoporphyrin IX on a Glioblastoma Cell Line. Pharmaceutics.

[208] Gallien J., Srinageshwar B., Gallo K., Holtgrefe G., Koneru S., Otero P.S., Bueno C.A., Mosher J., Roh A., Kohtz D.S. (2021). Curcumin Loaded Dendrimers Specifically Reduce Viability of Glioblastoma Cell Lines. Molecules.

[209] Sharma R., Liaw K., Sharma A., Jimenez A., Chang M., Salazar S., Amlani I., Kannan S., Kannan R.M. (2021). Glycosylation of PAMAM dendrimers significantly improves tumor macrophage targeting and specificity in glioblastoma. J. Control. Release.

[210] Uram L., Misiorek M., Pichla M., Filipowicz-Rachwal A., Markowicz J., Wolowiec S., Walajtys-Rode E. (2019). The Effect of Biotinylated PAMAM G3 Dendrimers Conjugated with COX-2 Inhibitor (celecoxib) and PPARgamma Agonist (Fmoc-L-Leucine) on Human Normal Fibroblasts, Immortalized Keratinocytes and Glioma Cells in Vitro. Molecules.

[211] Markowicz J., Wolowiec S., Rode W., Uram L. (2022). Synthesis and Properties of alpha-Mangostin and Vadimezan Conjugates with Glucoheptoamidated and Biotinylated 3rd Generation Poly(amidoamine) Dendrimer, and Conjugation Effect on Their Anticancer and Anti-Nematode Activities. Pharmaceutics.

[212] Lu Y., Han S., Zheng H., Ma R., Ping Y., Zou J., Tang H., Zhang Y., Xu X., Li F. (2018). A novel RGDyC/PEG co-modified PAMAM dendrimer-loaded arsenic trioxide of glioma targeting delivery system. Int. J. Nanomed..

[213] Sharma A.K., Gupta L., Sahu H., Qayum A., Singh S.K., Nakhate K.T., Ajazuddin, Gupta U. (2018). Chitosan Engineered PAMAM Dendrimers as Nanoconstructs for the Enhanced Anti-Cancer Potential and Improved In vivo Brain Pharmacokinetics of Temozolomide. Pharm. Res..

[214] Shi X., Ma R., Lu Y., Cheng Y., Fan X., Zou J., Zheng H., Li F., Piao J.G. (2020). iRGD and TGN co-modified PAMAM for multi-targeted delivery of ATO to gliomas. Biochem. Biophys. Res. Commun..

[215] Liu C., Zhao Z., Gao H., Rostami I., You Q., Jia X., Wang C., Zhu L., Yang Y. (2019). Enhanced blood-brain-barrier penetrability and tumor-targeting efficiency by peptide-functionalized poly(amidoamine) dendrimer for the therapy of gliomas. Nanotheranostics.

[216] Han S., Zheng H., Lu Y., Sun Y., Huang A., Fei W., Shi X., Xu X., Li J., Li F. (2018). A novel synergetic targeting strategy for glioma therapy employing borneol combination with angiopep-2-modified, DOX-loaded PAMAM dendrimer. J. Drug Target..

[217] Sahoo R.K., Kumar H., Jain V., Sinha S., Ajazuddin, Gupta U. (2023). Angiopep-2 Grafted PAMAM Dendrimers for the Targeted Delivery of Temozolomide: In Vitro and In Vivo Effects of PEGylation in the Management of Glioblastoma Multiforme. ACS Biomater. Sci. Eng..

[218] Liu F.H., Hou C.Y., Zhang D., Zhao W.J., Cong Y., Duan Z.Y., Qiao Z.Y., Wang H. (2018). Enzyme-sensitive cytotoxic peptide-dendrimer conjugates enhance cell apoptosis and deep tumor penetration. Biomater. Sci..

[219] Wu Y., Fan Q., Zeng F., Zhu J., Chen J., Fan D., Li X., Duan W., Guo Q., Cao Z. (2018). Peptide-Functionalized Nanoinhibitor Restrains Brain Tumor Growth by Abrogating Mesenchymal-Epithelial Transition Factor (MET) Signaling. Nano Lett..

[220] Ellert-Miklaszewska A., Ochocka N., Maleszewska M., Ding L., Laurini E., Jiang Y., Roura A.J., Giorgio S., Gielniewski B., Pricl S. (2019). Efficient and innocuous delivery of small interfering RNA to microglia using an amphiphilic dendrimer nanovector. Nanomedicine..

[221] Jin Z., Piao L., Sun G., Lv C., Jing Y., Jin R. (2021). Dual functional nanoparticles efficiently across the blood-brain barrier to combat glioblastoma via simultaneously inhibit the PI3K pathway and NKG2A axis. J. Drug Target..

[222] Sharma A., Liaw K., Sharma R., Thomas A.G., Slusher B.S., Kannan S., Kannan R.M. (2020). Targeting Mitochondria in Tumor-Associated Macrophages using a Dendrimer-Conjugated TSPO Ligand that Stimulates Antitumor Signaling in Glioblastoma. Biomacromolecules.

[223] Liaw K., Sharma R., Sharma A., Salazar S., Appiani La Rosa S., Kannan R.M. (2021). Systemic dendrimer delivery of triptolide to tumor-associated macrophages improves anti-tumor efficacy and reduces systemic toxicity in glioblastoma. J. Control. Release.

[224] Yazdani H., Kaul E., Bazgir A., Maysinger D., Kakkar A. (2020). Telodendrimer-Based Macromolecular Drug Design using 1,3-Dipolar Cycloaddition for Applications in Biology. Molecules.

[225] Tian M., Xing R., Guan J., Yang B., Zhao X., Yang J., Zhan C., Zhang S. (2021). A Nanoantidote Alleviates Glioblastoma Chemotoxicity without Efficacy Compromise. Nano Lett..

[226] Patil S., Mishra V.S., Yadav N., Reddy P.C., Lochab B. (2022). Dendrimer-Functionalized Nanodiamonds as Safe and Efficient Drug Carriers for Cancer Therapy: Nucleus Penetrating Nanoparticles. ACS Appl. Bio Mater..

[227] Muniswamy V.J., Raval N., Gondaliya P., Tambe V., Kalia K., Tekade R.K. (2019). ‘Dendrimer-Cationized-Albumin’ encrusted polymeric nanoparticle improves BBB penetration and anticancer activity of doxorubicin. Int. J. Pharm..

[228] Mishra V.S., Patil S., Reddy P.C., Lochab B. (2022). Combinatorial delivery of CPI444 and vatalanib loaded on PEGylated graphene oxide as an effective nanoformulation to target glioblastoma multiforme: In vitro evaluation. Front. Oncol..

[229] Soni K.S., Desale S.S., Bronich T.K. (2016). Nanogels: An overview of properties, biomedical applications and obstacles to clinical translation. J. Control. Release.

[230] Parkins C.C., McAbee J.H., Ruff L., Wendler A., Mair R., Gilbertson R.J., Watts C., Scherman O.A. (2021). Mechanically matching the rheological properties of brain tissue for drug-delivery in human glioblastoma models. Biomaterials.

[231] Voeikov R., Abakumova T., Grinenko N., Melnikov P., Bespalov V., Stukov A., Chekhonin V., Klyachko N., Nukolova N. (2017). Dioxadet-loaded nanogels as a potential formulation for glioblastoma treatment. J. Pharm. Investig..

[232] Chen W., Zou Y., Zhong Z., Haag R. (2017). Cyclo(RGD)-Decorated Reduction-Responsive Nanogels Mediate Targeted Chemotherapy of Integrin Overexpressing Human Glioblastoma In Vivo. Small.

[233] Liang Q., Zhuo Y., Wu X., Zheng S., Zhuang J., Wang K., Chen S. (2023). Curcumin combining temozolomide formed localized nanogel for inhibition of postsurgical chemoresistant glioblastoma. Nanomedicine.

[234] Godau B., Samimi S., Seyfoori A., Samiei E., Khani T., Naserzadeh P., Najafabadi A.H., Lesha E., Majidzadeh A.K., Ashtari B. (2023). A Drug-Eluting Injectable NanoGel for Localized Delivery of Anticancer Drugs to Solid Tumors. Pharmaceutics.

[235] Mandal P., Panja S., Banerjee S.L., Ghorai S.K., Maji S., Maiti T.K., Chattopadhyay S. (2020). Magnetic particle anchored reduction and pH responsive nanogel for enhanced intracellular drug delivery. Eur. Polym. J..

[236] Mandal P., Maji S., Panja S., Bajpai O.P., Maiti T.K., Chattopadhyay S. (2019). Magnetic particle ornamented dual stimuli responsive nanogel for controlled anticancer drug delivery. New J. Chem..

[237] Shatsberg Z., Zhang X., Ofek P., Malhotra S., Krivitsky A., Scomparin A., Tiram G., Calderón M., Haag R., Satchi-Fainaro R. (2016). Functionalized nanogels carrying an anticancer microRNA for glioblastoma therapy. J. Control. Release.

[238] Lopalco A., Cutrignelli A., Denora N., Perrone M., Iacobazzi R.M., Fanizza E., Lopedota A., Depalo N., de Candia M., Franco M. (2018). Delivery of Proapoptotic Agents in Glioma Cell Lines by TSPO Ligand-Dextran Nanogels. Int. J. Mol. Sci..

[239] Gao X., Li S., Ding F., Liu X., Wu Y., Li J., Feng J., Zhu X., Zhang C. (2021). A Virus-Mimicking Nucleic Acid Nanogel Reprograms Microglia and Macrophages for Glioblastoma Therapy. Adv. Mater..

[240] Yuan Y., Zhao H., Guo Y., Tang J., Liu C., Li L., Yao C., Yang D. (2020). A Programmable Hybrid DNA Nanogel for Enhanced Photodynamic Therapy of Hypoxic Glioma. Trans. Tianjin Univ..

[241] Li Q., Shen J., Wu L., Lei S., Yang Y., Xu W., Hao K., Zhang Y., Kong F., Yang W. (2023). Functional targeted therapy for glioma based on platelet membrane-coated nanogels. Cancer Nanotechnol..

[242] Zhang D., Tian S., Liu Y., Zheng M., Yang X., Zou Y., Shi B., Luo L. (2022). Near infrared-activatable biomimetic nanogels enabling deep tumor drug penetration inhibit orthotopic glioblastoma. Nat. Commun..

[243] Liu J., Li M., Huang Y., Zhang L., Li W., Cao P., Min W., Li J., Jing W. (2021). A Nanogel with Effective Blood-Brain Barrier Penetration Ability through Passive and Active Dual-Targeting Function. J. Nanomater..

[244] Singh S., Drude N., Blank L., Desai P.B., Konigs H., Rutten S., Langen K.J., Moller M., Mottaghy F.M., Morgenroth A. (2021). Protease Responsive Nanogels for Transcytosis across the Blood-Brain Barrier and Intracellular Delivery of Radiopharmaceuticals to Brain Tumor Cells. Adv. Healthc. Mater..

[245] Zhang M., Asghar S., Tian C., Hu Z., Ping Q., Chen Z., Shao F., Xiao Y. (2021). Lactoferrin/phenylboronic acid-functionalized hyaluronic acid nanogels loading doxorubicin hydrochloride for targeting glioma. Carbohydr. Polym..

[246] Baklaushev V.P., Nukolova N.N., Khalansky A.S., Gurina O.I., Yusubalieva G.M., Grinenko N.P., Gubskiy I.L., Melnikov P.A., Kardashova K., Kabanov A.V. (2015). Treatment of glioma by cisplatin-loaded nanogels conjugated with monoclonal antibodies against Cx43 and BSAT1. Drug Deliv..

[247] Nukolova N.V., Baklaushev V.P., Abakumova T.O., Mel’nikov P.A., Abakumov M.A., Yusubalieva G.M., Bychkov D.A., Kabanov A.V., Chekhonin V.P. (2014). Targeted delivery of cisplatin by small es, Cyrilliconnexin 43 vector nanogels to the focus of experimental glioma C6. Bull. Exp. Biol. Med..

[248] She D., Huang H., Li J., Peng S., Wang H., Yu X. (2021). Hypoxia-degradable zwitterionic phosphorylcholine drug nanogel for enhanced drug delivery to glioblastoma. Chem. Eng. J..

[249] Gadhave D., Rasal N., Sonawane R., Sekar M., Kokare C. (2021). Nose-to-brain delivery of teriflunomide-loaded lipid-based carbopol-gellan gum nanogel for glioma: Pharmacological and in vitro cytotoxicity studies. Int. J. Biol. Macromol..

[250] Iijima S. (1991). Helical microtubules of graphitic carbon. Nature.

[251] Saleemi M.A., Hosseini Fouladi M., Yong P.V.C., Chinna K., Palanisamy N.K., Wong E.H. (2021). Toxicity of Carbon Nanotubes: Molecular Mechanisms, Signaling Cascades, and Remedies in Biomedical Applications. Chem. Res. Toxicol..

[252] Eatemadi A., Daraee H., Karimkhanloo H., Kouhi M., Zarghami N., Akbarzadeh A., Abasi M., Hanifehpour Y., Joo S.W. (2014). Carbon nanotubes: Properties, synthesis, purification, and medical applications. Nanoscale Res. Lett..

[253] Benos L., Spyrou L.A., Sarris I.E. (2019). Development of a new theoretical model for blood-CNTs effective thermal conductivity pertaining to hyperthermia therapy of glioblastoma multiform. Comput. Methods Programs Biomed..

[254] Golubewa L., Kulahava T., Kunitskaya Y., Bulai P., Shuba M., Karpicz R. (2020). Enhancement of single-walled carbon nanotube accumulation in glioma cells exposed to low-strength electric field: Promising approach in cancer nanotherapy. Biochem. Biophys. Res. Commun..

[255] Nikeafshar S., Khazaei A., Tahvilian R. (2022). Inhibition of Methamphetamine-Induced Cytotoxicity in the U87-Cell Line by Atorvastatin-Conjugated Carbon Nanotubes. Appl. Biochem. Biotechnol..

[256] Hopkins S., Gottipati M.K., Montana V., Bekyarova E., Haddon R.C., Parpura V. (2018). Effects of Chemically-Functionalized Single-Walled Carbon Nanotubes on the Morphology and Vitality of D54MG Human Glioblastoma Cells. Neuroglia.

[257] Zhao D., Alizadeh D., Zhang L., Liu W., Farrukh O., Manuel E., Diamond D.J., Badie B. (2011). Carbon nanotubes enhance CpG uptake and potentiate antiglioma immunity. Clin. Cancer Res..

[258] Alizadeh D., White E.E., Sanchez T.C., Liu S., Zhang L., Badie B., Berlin J.M. (2018). Immunostimulatory CpG on Carbon Nanotubes Selectively Inhibits Migration of Brain Tumor Cells. Bioconjug Chem..

[259] Chen H., Shi Y., Sun L., Ni S. (2020). Electrospun composite nanofibers with all-trans retinoic acid and MWCNTs-OH against cancer stem cells. Life Sci..

[260] Wang X., Gong Z., Wang T., Law J., Chen X., Wanggou S., Wang J., Ying B., Francisco M., Dong W. (2023). Mechanical nanosurgery of chemoresistant glioblastoma using magnetically controlled carbon nanotubes. Sci. Adv..

[261] Salazar A., Perez-de la Cruz V., Munoz-Sandoval E., Chavarria V., Garcia Morales M.L., Espinosa-Bonilla A., Sotelo J., Jimenez-Anguiano A., Pineda B. (2021). Potential Use of Nitrogen-Doped Carbon Nanotube Sponges as Payload Carriers Against Malignant Glioma. Nanomater.

[262] Parikh S.D., Dave S., Huang L., Wang W., Mukhopadhyay S.M., Mayes D.A. (2020). Multi-walled carbon nanotube carpets as scaffolds for U87MG glioblastoma multiforme cell growth. Mater. Sci. Eng. C Mater. Biol. Appl..

[263] Minchenko D.O., Rudnytska O.V., Khita O.O., Kulish Y.V., Viletska Y.M., Halkin O.V., Danilovskyi S.V., Ratushna O.O., Minchenko O.H. (2023). Expression of DNAJB9 and some other genes is more sensitive to SWCNTs in normal human astrocytes than glioblastoma cells. Endocr. Regul..

[264] Janjua T.I., Cao Y., Ahmed-Cox A., Raza A., Moniruzzaman M., Akhter D.T., Fletcher N.L., Kavallaris M., Thurecht K.J., Popat A. (2023). Efficient delivery of Temozolomide using ultrasmall large-pore silica nanoparticles for glioblastoma. J. Control. Release.

[265] Janjua T.I., Ahmed-Cox A., Meka A.K., Mansfeld F.M., Forgham H., Ignacio R.M.C., Cao Y., McCarroll J.A., Mazzieri R., Kavallaris M. (2021). Facile synthesis of lactoferrin conjugated ultra small large pore silica nanoparticles for the treatment of glioblastoma. Nanoscale.

[266] Selvarajan V., Obuobi S., Ee P.L.R. (2020). Silica Nanoparticles-A Versatile Tool for the Treatment of Bacterial Infections. Front. Chem..

[267] Zhang Z., Zhao L., Ma Y., Liu J., Huang Y., Fu X., Peng S., Wang X., Yang Y., Zhang X. (2022). Mechanistic study of silica nanoparticles on the size-dependent retinal toxicity in vitro and in vivo. J. Nanobiotechnol..

[268] Kretowski R., Kusaczuk M., Naumowicz M., Cechowska-Pasko M. (2021). The Pro-Apoptotic Effect of Silica Nanoparticles Depends on Their Size and Dose, as Well as the Type of Glioblastoma Cells. Int. J. Mol. Sci..

[269] Zanoni D.K., Stambuk H.E., Madajewski B., Montero P.H., Matsuura D., Busam K.J., Ma K., Turker M.Z., Sequeira S., Gonen M. (2021). Use of Ultrasmall Core-Shell Fluorescent Silica Nanoparticles for Image-Guided Sentinel Lymph Node Biopsy in Head and Neck Melanoma: A Nonrandomized Clinical Trial. JAMA Netw. Open.

[270] Janjua T.I., Cao Y., Yu C., Popat A. (2021). Clinical translation of silica nanoparticles. Nat. Rev. Mater..

[271] Zhang H., Li M., Li J., Agrawal A., Hui H.W., Liu D. (2022). Superiority of Mesoporous Silica-Based Amorphous Formulations over Spray-Dried Solid Dispersions. Pharmaceutics.

[272] Shadmani N., Makvandi P., Parsa M., Azadi A., Nedaei K., Mozafari N., Poursina N., Mattoli V., Tay F.R., Maleki A. (2023). Enhancing Methotrexate Delivery in the Brain by Mesoporous Silica Nanoparticles Functionalized with Cell-Penetrating Peptide using in Vivo and ex Vivo Monitoring. Mol. Pharm..

[273] Sanchez-Dengra B., Alfonso M., Gonzalez-Alvarez I., Bermejo M., Gonzalez-Alvarez M., Martinez-Manez R. (2023). Intranasal administration of molecular-gated mesoporous nanoparticles to increase ponatinib delivery to the brain. Nanomedicine.

[274] Shahein S.A., Aboul-Enein A.M., Higazy I.M., Abou-Elella F., Lojkowski W., Ahmed E.R., Mousa S.A., AbouAitah K. (2019). Targeted anticancer potential against glioma cells of thymoquinone delivered by mesoporous silica core-shell nanoformulations with pH-dependent release. Int. J. Nanomed..

[275] Chavarria V., Ortiz-Islas E., Salazar A., Perez-de la Cruz V., Espinosa-Bonilla A., Figueroa R., Ortiz-Plata A., Sotelo J., Sanchez-Garcia F.J., Pineda B. (2022). Lactate-Loaded Nanoparticles Induce Glioma Cytotoxicity and Increase the Survival of Rats Bearing Malignant Glioma Brain Tumor. Pharmaceutics.

[276] Zhu J., Zhang Y., Chen X., Zhang Y., Zhang K., Zheng H., Wei Y., Zheng H., Zhu J., Wu F. (2021). Angiopep-2 modified lipid-coated mesoporous silica nanoparticles for glioma targeting therapy overcoming BBB. Biochem. Biophys. Res. Commun..

[277] Tao J., Fei W., Tang H., Li C., Mu C., Zheng H., Li F., Zhu Z. (2019). Angiopep-2-Conjugated “Core-Shell” Hybrid Nanovehicles for Targeted and pH-Triggered Delivery of Arsenic Trioxide into Glioma. Mol. Pharm..

[278] Juthani R., Madajewski B., Yoo B., Zhang L., Chen P.M., Chen F., Turker M.Z., Ma K., Overholtzer M., Longo V.A. (2020). Ultrasmall Core-Shell Silica Nanoparticles for Precision Drug Delivery in a High-Grade Malignant Brain Tumor Model. Clin. Cancer Res..

[279] Sapre A.A., Yong G., Yeh Y.S., Ruff L.E., Plaut J.S., Sayar Z., Agarwal A., Martinez J., Nguyen T.N., Liu Y.T. (2019). Silica cloaking of adenovirus enhances gene delivery while reducing immunogenicity. J. Control. Release.

[280] Feng Y., Cao Y., Qu Z., Janjua T.I., Popat A. (2023). Virus-like Silica Nanoparticles Improve Permeability of Macromolecules across the Blood-Brain Barrier In Vitro. Pharmaceutics.

[281] Zhang P., Tang M., Huang Q., Zhao G., Huang N., Zhang X., Tan Y., Cheng Y. (2019). Combination of 3-methyladenine therapy and Asn-Gly-Arg (NGR)-modified mesoporous silica nanoparticles loaded with temozolomide for glioma therapy in vitro. Biochem. Biophys. Res. Commun..

[282] Chen Z.A., Wu C.H., Wu S.H., Huang C.Y., Mou C.Y., Wei K.C., Yen Y., Chien I.T., Runa S., Chen Y.P. (2024). Receptor Ligand-Free Mesoporous Silica Nanoparticles: A Streamlined Strategy for Targeted Drug Delivery across the Blood-Brain Barrier. ACS Nano.

[283] Li M., Cui X., Wei F., Li C., Han X. (2022). RGD Peptide Modified Erythrocyte Membrane/Porous Nanoparticles Loading Mir-137 for NIR-Stimulated Theranostics of Glioblastomas. Nanomaterials.

[284] Turan O., Bielecki P.A., Perera V., Lorkowski M., Covarrubias G., Tong K., Yun A., Loutrianakis G., Raghunathan S., Park Y. (2019). Treatment of glioblastoma using multicomponent silica nanoparticles. Adv Ther.

[285] Wu M., Zhang H., Tie C., Yan C., Deng Z., Wan Q., Liu X., Yan F., Zheng H. (2018). MR imaging tracking of inflammation-activatable engineered neutrophils for targeted therapy of surgically treated glioma. Nat. Commun..

[286] Prabhakar N., Merisaari J., Le Joncour V., Peurla M., Karaman D.S., Casals E., Laakkonen P., Westermarck J., Rosenholm J.M. (2021). Circumventing Drug Treatment? Intrinsic Lethal Effects of Polyethyleneimine (PEI)-Functionalized Nanoparticles on Glioblastoma Cells Cultured in Stem Cell Conditions. Cancers.

[287] Nikolopoulou S.G., Kalska B., Basa A., Papadopoulou A., Efthimiadou E.K. (2023). Novel Hybrid Silver-Silica Nanoparticles Synthesized by Modifications of the Sol-Gel Method and Their Theranostic Potential in Cancer. ACS Appl. Bio Mater..

[288] Ferreira L.M., Azambuja J.H., da Silveira E.F., Marcondes Sari M.H., da Cruz Weber Fulco B., Costa Prado V., Gelsleichter N.E., Beckenkamp L.R., da Cruz Fernandes M., Spanevello R.M. (2019). Antitumor action of diphenyl diselenide nanocapsules: In vitro assessments and preclinical evidence in an animal model of glioblastoma multiforme. J. Trace Elem. Med. Biol..

[289] Xu B., Zhang Q., Luo X., Ning X., Luo J., Guo J., Liu Q., Ling G., Zhou N. (2020). Selenium nanoparticles reduce glucose metabolism and promote apoptosis of glioma cells through reactive oxygen species-dependent manner. Neuroreport.

[290] Abadi B., Khazaeli P., Forootanfar H., Ranjbar M., Ahmadi-Zeidabadi M., Nokhodchi A., Ameri A., Adeli-Sardou M., Amirinejad M. (2023). Chitosan-sialic acid nanoparticles of selenium: Statistical optimization of production, characterization, and assessment of cytotoxic effects against two human glioblastoma cell lines. Int. J. Pharm..

[291] Turovsky E.A., Varlamova E.G. (2021). Mechanism of Ca(2+)-Dependent Pro-Apoptotic Action of Selenium Nanoparticles, Mediated by Activation of Cx43 Hemichannels. Biology.

[292] Varlamova E.G., Goltyaev M.V., Mal’tseva V.N., Turovsky E.A., Sarimov R.M., Simakin A.V., Gudkov S.V. (2021). Mechanisms of the Cytotoxic Effect of Selenium Nanoparticles in Different Human Cancer Cell Lines. Int. J. Mol. Sci..

[293] Mazarei M., Mohammadi Arvejeh P., Mozafari M.R., Khosravian P., Ghasemi S. (2021). Anticancer Potential of Temozolomide-Loaded Eudragit-Chitosan Coated Selenium Nanoparticles: In Vitro Evaluation of Cytotoxicity, Apoptosis and Gene Regulation. Nanomaterials.

[294] Varlamova E.G., Khabatova V.V., Gudkov S.V., Turovsky E.A. (2023). Ca(2+)-Dependent Effects of the Selenium-Sorafenib Nanocomplex on Glioblastoma Cells and Astrocytes of the Cerebral Cortex: Anticancer Agent and Cytoprotector. Int. J. Mol. Sci..

[295] Song Z., Liu T., Chen T. (2018). Overcoming blood-brain barrier by HER2-targeted nanosystem to suppress glioblastoma cell migration, invasion and tumor growth. J. Mater. Chem. B.

[296] Tang X., Wang Z., Xie Y., Liu Y., Yang K., Li T., Shen H., Zhao M., Jin J., Xiao H. (2023). Radiation-Triggered Selenium-Engineered Mesoporous Silica Nanocapsules for RNAi Therapy in Radiotherapy-Resistant Glioblastoma. ACS Nano.

[297] Ashokan A., Sarkar S., Kamran M.Z., Surnar B., Kalathil A.A., Spencer A., Dhar S. (2024). Simultaneous targeting of peripheral and brain tumors with a therapeutic nanoparticle to disrupt metabolic adaptability at both sites. Proc. Natl. Acad. Sci. USA.

[298] Yagublu V., Karimova A., Hajibabazadeh J., Reissfelder C., Muradov M., Bellucci S., Allahverdiyev A. (2022). Overview of Physicochemical Properties of Nanoparticles as Drug Carriers for Targeted Cancer Therapy. J. Funct. Biomater..

